# Factor Analysis Procedures Revisited from the Comprehensive Model with Unique Factors Decomposed into Specific Factors and Errors

**DOI:** 10.1007/s11336-021-09824-8

**Published:** 2022-02-01

**Authors:** Kohei Adachi

**Affiliations:** Osaka University, 1-2 Yamadaoka, Suita, Osaka 565-0871 Japan

**Keywords:** comprehensive factor analysis model, matrix decomposition factor analysis, completely decomposed factor analysis, latent variable factor analysis, Inter-variable error correlations

## Abstract

Factor analysis (FA) procedures can be classified into three types (Adachi in WIREs Comput Stat https://onlinelibrary.wiley.com/doi/abs/10.1002/wics.1458, 2019): latent variable FA (LVFA), matrix decomposition FA (MDFA), and its variant (Stegeman in Comput Stat Data Anal 99: 189–203, 2016) named completely decomposed FA (CDFA) through the theorems proved in this paper. We revisit those procedures from the Comprehensive FA (CompFA) model, in which a multivariate observation is decomposed into common factor, specific factor, and error parts. These three parts are separated in MDFA and CDFA, while the specific factor and error parts are not separated, but their sum, called a unique factor, is considered in LVFA. We show that the assumptions in the CompFA model are satisfied by the CDFA solution, but not completely by the MDFA one. Then, how the CompFA model parameters are estimated in the FA procedures is examined. The study shows that all parameters can be recovered well in CDFA, while the sum of the parameters for the specific factor and error parts is approximated by the LVFA estimate of the unique factor parameter and by the MDFA estimate of the specific factor parameter. More detailed results are given through our subdivision of the CompFA model according to whether the error part is uncorrelated among variables or not.

Factor analysis (FA) was originally conceived of by Spearman (1904) and developed toward its modern form by Thurstone (1947). FA is performed for a multivariate data set in order to extract two types of mutually uncorrelated factors: common factors and all others. The common factors, whose number is much less than that of observed variables, serve to explain the variation of all variables. On the other hand, the other factors explain the variations in the variables that remain unaccounted for by the common factors. The latter factors are referred to as unique factors in a prevalent FA model, while those factors are called specific factors in another FA model. Those two FA models are introduced in the following two paragraphs.

The prevalent FA model is expressed as

1 $$\begin{aligned} \mathbf{x }= {\varvec{\Lambda }} \mathbf{f } +{{\tilde{\mathbf{e}}}}= {\varvec{\Lambda }} \mathbf{f } + {\varvec{\Psi }} \mathbf{u }^{\mathbf {\, }} \end{aligned}$$

for a *p*-variate observed vector **x**(*p* $\times$ 1), with ${{\tilde{\mathbf{e}}}}=$ ${\varvec{\Psi }}\mathbf{u }$ and the expectations of the elements in **x** being zero (e.g., Bartholomew, Knott, & Moustaki, 2011; Mulaik, 2010; Yanai & Ichikawa, 2007). Here, vectors $\mathbf{f }\,(m\times 1)$ and $\mathbf{u }\,(p\times 1)$ contain the common and unique factor scores, respectively, with *m* < *p*. The scores in **f** and **u** are treated as random latent variables, while $\varvec{\Lambda }$ ($p\times m)$ and ${\varvec{\Psi }}(p\times p)$ are the nonrandom parameter matrices to be estimated. The elements of $\varvec{\Lambda }$ are called factor loadings, as they describe how the *p* variables in **x** load the *m* common factors in **f**. In contrast to $\varvec{\Lambda }$ being unconstrained, $\varvec{\Psi }$ is restricted to a diagonal matrix. This implies that the *p* variables in **x** have one-to-one correspondences to the *p* unique factors in **u**: its *j*th element uniquely affects the *j*th one of **x** with the *j*th diagonal element of $\varvec{\Psi }$ being a coefficient. We refer to the FA procedure based on (1) as *latent variable FA* (*LVFA*) following Adachi (2019), in order to distinguish it from a procedure to be introduced later.

The vector ${{\tilde{\mathbf{e}}}}={\varvec{\Psi }}\mathbf{u }$ in (1) is sometimes referred to as an error vector; however, the classic literature such as Harman (1976), Reyment & Jöreskog (1993), and Thurstone (1947) includes the description that ${{\tilde{\mathbf{e}}}}= {\varvec{\Psi }}\mathbf{u }$ is divided into two vectors as

2 $$\begin{aligned} {\varvec{\Psi }} \mathbf{u } ={\varvec{\Theta }} \mathbf{s }+ \mathbf{e } . \end{aligned}$$

Here, **e** (rather than ${{\tilde{\mathbf{e}}}})$ is referred to as an error vector, and the elements of **s** (*p* $\times$ 1) are called specific factor scores, with $\varvec{\Theta }$ (*p* $\times$ *p*) being diagonal. That is, the FA model is also introduced by incorporating (2) into (1), i.e.,

3 $$\begin{aligned} \mathbf{x } = {\varvec{\Lambda }} \mathbf{f } + {\varvec{\Theta }} \mathbf{s }+ \mathbf{e }.^{\mathbf {\, }} \end{aligned}$$

Here, $\varvec{\Theta }\mathbf{s }$ is found to perform a role similar to the unique factor part $\varvec{\Psi }\mathbf{u }$ in (1): $\varvec{\Theta }$ is diagonal as is $\varvec{\Psi }$, which implies that the *j*th specific factor score in **s** specifically (i.e., uniquely) affects the *j*th variable of **x**, with the *j*th diagonal element of $\varvec{\Theta }$ a coefficient. As found in this sentence, the adjective "specific" used for **s** in (3) has the same implication as the "unique" for **u** in (1): "specific" and "unique" merely serve to avoid the confusion between ${\varvec{\Psi }}\mathbf{u }$ in (1) and ${\varvec{\Theta }}\mathbf{s }$ in (3). However, the error vector **e** is included in (3) in addition to ${\varvec{\Theta }}\mathbf{s }$, while the error vector ${{\tilde{\mathbf{e}}}}$ in (1) equals ${\varvec{\Psi }}$ **u**. That is, each error in ${{\tilde{\mathbf{e}}}}$ is assumed to uniquely affect the corresponding variable in (1), but ${\varvec{\Theta }}\mathbf{s }$ is separated from **e**. In this point, (3) is more generalized or comprehensive than (1). We thus refer to (3) as a *comprehensive FA* (*CompFA*) *model*. This model can also be considered compatible with Spearman’s (1904) original conception of FA (Anderson & Rubin, 1956, p. 112; Yanai, Shegemasu, Mayekawa, & Ichikawa, 1990, p. 4).

However, the CompFA model has been left out of consideration in recent FA studies, as found in the fact that (3) is not treated in the major introductions to FA published in this century (e.g., Bartholomew, Knott, & Moustaki, 2011; Mulaik, 2010). That can be attributed to the following two points seen in the above classic literature: First, the CompFA model has been very briefly mentioned on only a couple of pages in Harman (1976, pp. 19–20), Reyment and Jöreskog, (1993, pp. 75–76), and Thurstone (1947, pp. 74–75); thus, model (3) was not impressed as fulfilling a certain role in FA. Second, in that literature, (3) has been introduced merely as a model; no procedure is described for estimating parameters based on (3).

However, a recently proposed FA procedure, which is called matrix decomposition FA (MDFA) to distinguish it from LVFA (Adachi, 2019; Adachi & Trendafilov, 2018), can be viewed as a parameter estimation procedure for the CompFA model. In more exactness, MDFA can be modeled as a nonrandom matrix version of (3):

4 $$\begin{aligned} {\mathbf{X}} = {\mathbf{F}}{\varvec{\Lambda }}^{\prime } + \mathbf{S }{\varvec{\Theta }} + \mathbf{E }.^{\mathbf {\, }} \end{aligned}$$

Here, **X** is an *n*-observations $\times$ *p*-variables column-centered data matrix, **F** is the *n* $\times$ *m* matrix of common factor scores, **S** is the *n* $\times$ *p* matrix of specific factor scores, and **E** is an *n* $\times$ *p* error matrix, with ${\varvec{\Lambda }}$ and ${\varvec{\Theta }}$ being the same as those in (3). The rows of **X**, **F**, **S**, and **E** in (4) correspond to the transposes of **x**, **f**, **s**, and **e** in (3), respectively. However, **F** and **S** in (4) are treated as nonrandom parameter matrices, while **f** and **s** in (3) are random variable vectors. To the best of our knowledge, MDFA was first proposed by Professor Henk A. L. Kiers at the University of Groningen in 2001, as described in Sočan (2003, pp. 19–20). Then, the properties of the MDFA solutions were studied by Adachi and Trendafilov (2018) and Stegeman (2016). The last author has also proposed a restrictive variant of MDFA. The factor vectors **f** and **u** in LVFA model (1) are random, as are **f** and **s** in (3), but **F** and **S** in MDFA model (4) are not random, as described above. This difference is not crucial in this paper, but rather we note that the unique factor part in (1) is decomposed into the specific factor and error parts in (3), whose nonrandom matrix version is (4).

In the above MDFA papers, the elements in the matrix corresponding to **S** have been referred to as unique factor scores, but they must be called specific factor scores according to the terminology in CompFA model (4). Such a confusing reference to **S** in the MDFA papers is due to the fact that the CompFA model was not related to MDFA there. This fact provides a major motivation for this paper: we study the relationships of the CompFA model to MDFA and its restrictive variant. This study includes reformulating those MDFA procedures to elucidate whether their parameter estimates are matched to the CompFA model assumptions for the parameters, with those assumptions to be introduced in Sect. 1. Here, we must inform beforehand that the reformulation (in Sect. 4) would allow Stegeman’s (2016) restrictive variant of MDFA to be rephrased as *completely decomposed FA* (*CDFA*). We thus call the variant CDFA hereafter. The relationships of LVFA to the CompFA model are also studied in this paper, as model (1) for LVFA is linked to the CompFA model through (2). The goal of our studies is to theoretically and empirically show how LVFA, MDFA, and CDFA behave for CompFA data, i.e., how the parameters in the CompFA model are recovered in the FA procedures, where the CompFA data refer to the data underlaid by the CompFA model. The goal would include showing that all parameters in that model can be recovered fairly well in CDFA but cannot be recovered as well in LVFA and MDFA.

The remaining sections in this paper are organized as follows: In Sect. 1, we specify the CompFA model by introducing its assumptions. Then, LVFA, MDFA, and CDFA are treated in Sects. 2,  3, and 4, respectively; we theoretically discuss how each procedure is related to the CompFA model and behaves for the CompFA data. The discussions for the behaviors are numerically assessed in Sect. 5 and illustrated in Sect. 6. Throughout this paper, we suppose *n* > *p* > *m*.

A more detailed prospect for the remaining sections will be presented in the final part of the next section, as that presentation is possible only after the CompFA model is fully specified. This specification also includes our subdivision of the CompFA model along whether the errors are assumed to be uncorrelated among variables or not. The subdivision is made, because the behaviors of the FA procedures differ between the CompFA data with uncorrelated errors and those with correlated ones, as discussed in Sects. 2, 3, 4 and shown numerically in Sect. 5.

## Comprehensive Factor Analysis Model

In Sects. 1.1 and 1.2, we review the standard assumptions for CompFA model (3) and its nonrandom matrix version (4), respectively. Those standard assumptions are not involved with the inter-variable correlations of errors. We discuss in Sect. 1.3 that the consideration of the error correlations allows the CompFA model to be subdivided. The prospects for the following sections are given in the final subsection.

### Random Version of Standard Assumptions

The CompFA model is expressed as (3), i.e., $\mathbf{x }={\varvec{\Lambda }}\mathbf{f }+{\varvec{\Theta }}\mathbf{s }+\mathbf{e }$, for the random observation vector **x**, whose expectation *E*[**x**] is the $p\times 1$ zero vector **0**$_{p}$. We review the assumptions for the expectations and covariances of **f**, **s**, and **e** in the classic literature (Harman, 1976; Reyment & Jöreskog, 1993; Thurstone, 1947).

In line with $E[\mathbf{x }]=\mathbf{0 }_{p}$, the expectations of the common factor, specific factor, and error vectors are supposed as

5 $$\begin{aligned} E[\mathbf{f }] = \mathbf{0 }_{m}, E[\mathbf{s }] = \mathbf{0 }_{p}, E[\mathbf{e }] = \mathbf{0 }_{p} . \end{aligned}$$

The covariance matrices for the factor score vectors are assumed to satisfy

6 $$\begin{aligned} C[\mathbf{f },\mathbf{f }] = \mathbf{I }_{m}, C[\mathbf{s },\mathbf{s }] = \mathbf{I }_{p}, C[\mathbf{f },\mathbf{s }] ={_m\mathbf{O }}_{p} . \end{aligned}$$

Here, *C*[**f**, **s**] $=$ *E*[(**f**$-E$[**f**])(**s **$-E$[**s**])$^\prime$] denotes the *m* $\times$ *p* covariance matrix between **f** and **s**, **I**$_{m\, }$ is the *m* $\times$ *m* identity matrix, and $_{m}$ **O**$_{p}$ expresses the *m* $\times$ *p* zero matrix. The factor score vectors are assumed to be uncorrelated to the error vector:

7 $$\begin{aligned} C[\mathbf{f },\mathbf{e }] ={_m\mathbf{O }}_{p}, C[\mathbf{s },\mathbf{e }] ={_p\mathbf{O }}_{\!p} . \end{aligned}$$

The standard constraints for model (3) consist of (5)–(7).

From (6), the covariance matrix of ${\varvec{\Lambda }}\mathbf{f }$ and that of ${\varvec{\Theta }}$ **s** are found to be $C[{\varvec{\Lambda }}\mathbf{f },{\varvec{\Lambda }}\mathbf{f }]={\varvec{\Lambda }}{C}[\mathbf{f }, \mathbf{f }]{\varvec{\Lambda }}^{\prime } = {\varvec{\Lambda }}{\varvec{ \Lambda }} ^\prime$ and $C[{\varvec{\Theta }} \mathbf{s }, {\varvec{\Theta }} \mathbf{s }] = {\varvec{\Theta }} C[\mathbf{s }, \mathbf{s }] {\varvec{\Theta }}^{\prime } ={\varvec{\Theta }}^{2}$, respectively. Here, the diagonal elements of ${\varvec{\Theta }}^{2\, }$ are called specific variances, as they stand for the variances of the specific factor part ${\varvec{\Theta }}$ **s**. Further, (6) and (7) lead to $C[{\varvec{\Lambda }} \mathbf{f }, {\varvec{\Theta }}\mathbf{s }] = C[{\varvec{\Lambda }} \mathbf{f },\mathbf{e }] ={_m\mathbf{O }}_{p}$ and $C[{\varvec{\Theta }} \mathbf{s }, \mathbf{e }] ={_p\mathbf{O }}_{\!p}$. Using these results, the inter-variable covariance matrix $C[\mathbf{x },\mathbf{x }]$ for (3) is found to be expressed as

8 $$\begin{aligned} C[\mathbf{x }, \mathbf{x }] = C[ {\varvec{\Lambda }} \mathbf{f } + {\varvec{\Theta }} \mathbf{s }+ \mathbf{e }, {\varvec{\Lambda }} \mathbf{f } + {\varvec{\Theta }} \mathbf{s }+ \mathbf{e }] = {\varvec{\Lambda }} {\varvec{\Lambda }}^{\prime } + {\varvec{\Theta }}^{2\, }+ C[\mathbf{e }, \mathbf{e }] . \end{aligned}$$

### Nonrandom Matrix Version of Standard Assumptions

The nonrandom matrix version of the CompFA model can be expressed as (4), i.e., $\mathbf{X } =\mathbf{F }{\varvec{\Lambda }}^{\prime } +\mathbf{S }{\varvec{\Theta }} + \mathbf{E }$ for the $n\times p$ data matrix **X**. Here, **X** is column-centered with **1**$_{n}^\prime \mathbf{X } =\mathbf{0 }_{p}^\prime$ and supposed to have full column rank with rank(**X**) $= p$, with **1**$_{n}$ and rank(**X**) denoting the *n* $\times$ 1 vector of ones and the rank of **X**, respectively. We summarize the assumptions for (4), i.e., the matrix versions of the assumptions in Sect. 1.1.

The versions of (5) and (6) are expressed as

9 $$\begin{aligned}&\mathbf{1 }_{n}^\prime {\mathbf{F}} = \mathbf{0 }_{m}^\prime , \mathbf{1 }_{n}^\prime \mathbf{S } = \mathbf{0 }_{p}^\prime , \mathbf{1 }_{n}^\prime \mathbf{E } = \mathbf{0 }_{p}^\prime , \end{aligned}$$

10 $$\begin{aligned}&\frac{1}{n}{\mathbf{F}}^\prime {\mathbf{F}} = \mathbf{I }_{m} , \frac{1}{n}\mathbf{S }^\prime \mathbf{S } = \mathbf{I }_{p\, }, \frac{1}{n}{\mathbf{F}}^\prime \mathbf{S } = {}_{m}\mathbf{O }_{p}\,\, \hbox {or}\,\, {\mathbf{F}}^\prime \mathbf{S } = {}_{m}\mathbf{O }_{p} , \end{aligned}$$

respectively. The two equations in (7) can be changed into the matrix forms $n^{-1}\mathbf{F }^\prime \mathbf{E }=\,{{}_m\mathbf{O }}_{p}$ and $n^{-1}\mathbf{S }^\prime \mathbf{E }=\,{{}_p\mathbf{O }}_{\!p}$,which are equivalent to

11 $$\begin{aligned} {\mathbf{F}}^\prime \mathbf{E }=\,{}_{m}\mathbf{O }_{p}, \end{aligned}$$

12 $$\begin{aligned} \mathbf{S }^\prime \mathbf{E }=\,{}_{p}\mathbf{O }_{p} , \end{aligned}$$

respectively. The standard assumptions for model (4) consist of (9)–(12).

The $p\times p$ inter-variable matrices for **X** and **E** can be expressed as $\mathbf{C }_{\mathrm{XX}} = n^{-1}\mathbf{X }^\prime \mathbf{X }$ and $\mathbf{C }_{\mathrm{EE}}= n^{-1}\mathbf{E }^\prime \mathbf{E }$, because of $\mathbf{1 }_{n}{}^\prime \mathbf{X } =\mathbf{0 }_{p}{}^\prime$ and (9). Then, (9)–(12) lead to the nonrandom matrix version of (8):

13 $$\begin{aligned} \mathbf{C }_{\mathrm{XX}} =\frac{1}{n}({\mathbf{F}}{\varvec{\Lambda }}^{\prime } + \mathbf{S }{\varvec{\Theta }} + \mathbf{E })^\prime ({\mathbf{F}}{\varvec{\Lambda }}^{\prime } + \mathbf{S }{\varvec{\Theta }} + \mathbf{E }) = {\varvec{\Lambda }}{\varvec{ \Lambda }}^{\prime } + {\varvec{\Theta }}^{2\, }+ \mathbf{C }_{\mathrm{EE}} , \end{aligned}$$

where **F**${\varvec{\Lambda }}^{\prime } +$ **S**${\varvec{\Theta }}$ $+$ **E** is column-centered because of (9).

### Uncorrelated Error and Correlated Error Assumptions

In the classic literature (Harman, 1976; Reyment & Jöreskog 1993; Thurstone, 1947), the elements of **e** in (3) are particularly referred to as measurement errors. If such errors are considered to be uncorrelated among variables, we can add the assumption that the off-diagonal elements of *C*[**e**, **e**] are zeros, i.e.,

14 $$\begin{aligned} C[\mathbf{e }, \mathbf{e }] = D[\mathbf{e }, \mathbf{e }] \end{aligned}$$

to those in Sect. 1.1, with *D*[**e**, **e**] $=$ diag(*C*[**e**, **e**]). Here, diag(**N**) denotes the diagonal matrix whose diagonal elements are those of a square matrix **N**. In the classic literature, (14) is not explicitly presented, but might be implicitly supposed for the following reasons: In that literature, only latent variable FA (LVFA) is described for estimating parameters, and the constraints considered in LVFA follow from adding (14) to the standard ones in Sect. 1.1, as shown in Sect. 2.2. We can also add the nonrandom matrix version of (14), i.e.,

15 $$\begin{aligned} \mathbf{C }_{\mathrm{EE}}= \mathbf{D }_{\mathrm{EE}} \end{aligned}$$

to those in Sect. 1.2, with **D**$_{\mathrm{EE}} =$ diag(**C**$_{\mathrm{EE}})$.

We can also consider a version of the CompFA model, in which (14) and (15) are not assumed, that is, the errors are allowed to be correlated among variables. In this correlated error version, *C*[**e**, **e**] in (8) and **C**$_{\mathrm{EE}}$ in (13) are merely supposed to be unconstrained covariance matrices that are nonnegative-definite.

We should note the difference between uncorrelated error constraints (14) and (15), the latter being stronger, as explained next. The rows of the error matrix **E** in **C**$_{\mathrm{EE}}=$ $n^{-1}$ **E**$^\prime$ **E** can be considered the realizations of the transpose of **e** in (14). This consideration leads to *C*[**e**, **e**] in (14) equaling *E*[**C**$_{\mathrm{EE}}$], as detailed in Appendix 1. Thus, we can rewrite (14) as the expectation of **C**$_{\mathrm{EE}}$ being diagonal: (14) requires the diagonality of *E*[**C**$_{\mathrm{EE}}$], but not that of **C**$_{\mathrm{EE}}$ itself. This diagonality is required by (15), in contrast. To emphasize the strength of (15), we call this the *strong* uncorrelated error condition. We can consider that data meeting (15) is hardly encountered, i.e., is unusual. However, in the later sections, such data would be noted: The FA procedures are shown to perfectly fit the data meeting strong condition (15), which is a motivation to study the related properties of the FA procedures. The next theorem gives the foundation for the perfect fit to be shown later.

#### Theorem 1

If the error matrix  in CompFA model (4) satisfies (15), (4) can be rewritten as the error-free model:

16 $$\begin{aligned} {\mathbf{X}}= {\mathbf{F}}{\varvec{\Lambda }}^{\prime } +{{\tilde{\mathbf{S}}\tilde{\varvec{\Theta }}}}= {\mathbf{F}}{\varvec{\Lambda }}^{\prime } +{{\tilde{\mathbf{S}}}}({\varvec{\Theta }}^{2}+{\mathbf{D}}_{\mathrm{EE}} )^{1/2}. \end{aligned}$$

Here, ${\varvec{\tilde{{\Theta }}}}^{2}=$ ${\varvec{\Theta }}^{2\, }+$ **D**$_{\mathrm{EE}}$ and ${{\tilde{\mathbf{S}}}} =$(**S**${\varvec{\Theta }}$ + **E**)${\varvec{\tilde{{\Theta }}}}^{-1}$ can be regarded as the specific variance and factor score matrices, respectively.

#### Proof

We can rewrite (4) as (16): **X**$=$ **F**${\varvec{\Lambda }}^{\prime } +$ **S**${\varvec{\Theta }} +$ **E** $=$ **F**${\varvec{\Lambda }}^{\prime } +$ (**S**${\varvec{\Theta }} +$ **E**)${\varvec{\tilde{{\Theta }}}}^{-1}{\varvec{\tilde{{\Theta }}}}$. The matrix ${\varvec{\tilde{{\Theta }}}}^{2}=$ ${\varvec{\Theta }}^{2\, }+$ **D**$_{\mathrm{EE}}$ (*p* $\times$ *p*) is diagonal and nonnegative, thus regarded as the specific variance matrix. We can show why ${{\tilde{\mathbf{S}}}} =$ (**S**${\varvec{\Theta }}$ $+$ **E**)${\varvec{\tilde{{\Theta }}}}^{-1}(n$ $\times$ *p*) is the specific factor score matrix, as follows: (9)–(12) and (15) lead to ${{\mathbf{1}}}_{n}{}^\prime {{\tilde{\mathbf{S}}}}=$ **1**$_{n}{}^\prime$(**S**${\varvec{\Theta }}$ $+$ **E**)${\varvec{\tilde{{\Theta }}}}^{-1}=$ **0**$_{p}{}^\prime$,

$$\begin{aligned} \frac{1}{n}{{{\tilde{\mathbf{S }}}^\prime \tilde{{{\mathbf{S}}}}}} = \frac{1}{n} {\varvec{\tilde{{\Theta }}}}^{-1}({\mathbf{S}}{\varvec{\Theta }} + {\mathbf{E}})^{\prime } (\mathbf{S }{\varvec{\Theta }} + \mathbf{E }){\varvec{\tilde{{\Theta }}}}^{-1}= ( \varvec{\Theta }^{2\, }+ \mathbf{D }_{\mathrm{EE}})^{-1/2}( \varvec{\Theta } ^{2\, }+\mathbf{D }_{\mathrm{EE}}) ( \varvec{\Theta }^{2\, }+ \mathbf{D }_{\mathrm{EE}})^{-1/2}= \mathbf{I }_{p} , \end{aligned}$$

and ${{{\mathbf{F}}^{\prime }\tilde{{\mathbf{S}}}}}=$ $\mathbf{F} ^{\prime }$(**S**$\varvec{\Theta }$ $+$ **E**)${\varvec{\tilde{{\Theta }}}}^{-1}=$ $_{m}$ **O**$_{p}$. Thus, ${{\tilde{{\mathbf{S}}}}} =$ (**S**$\varvec{\Theta }$ $+$ **E**)${\varvec{\tilde{{\Theta }}}}^{-1}$ can be substituted into **S** in (9)–(11). Further, (16) can be rewritten as **X**$=$ **F**${\varvec{\Lambda }}^\prime +{{\tilde{{\mathbf{S}}}\tilde{\varvec{\Theta }}}}+{{\tilde{{\mathbf{E}}}}}$, with the error matrix ${{\tilde{{\mathbf{E}}}}}$ being $_{n}\mathbf{O }_{p}$, which implies that those ${{\tilde{{\mathbf{E}}}}}$ and ${{\tilde{{\mathbf{S}}}}}$ can be substituted into **E** and **S** in (12), respectively. $\square$

### Prospects for Relating the CompFA Model to LVFA, MDFA, and CDFA

As the assumptions in the CompFA model have been specified, they can now be used for providing the prospects for the following sections, where the relationships of the CompFA model to LVFA, MDFA, and CDFA will be studied.

Among the relationships, those independent of the uncorrelated and correlated error assumptions (in Sect. 1.3) can be summarized as in Table 1. Its left-hand "Model" and "Note" columns merely present the facts described before Sect. 1: (2) links LVFA model (1) to CompFA model (3), and this matrix version is model (4) underlying MDFA and CDFA.

The right-hand columns in Table 1 present the key points in the relationships to be found. The lower cells in the "Specific Factor & Errors" column show the facts to be found in Sects. 3.2 and 4.2: The MDFA solution does not meet (12) in the CompFA assumptions, but only satisfies its diagonal part diag(**S**$^\prime$ **E**) $=$ $_{p}$ **O**$_{p}$, but (12) is satisfied by the CDFA solution. On the other hand, the specific factor and error parts ($\varvec{\Theta }\,$ **s** and **e**) are unseparated in LVFA.

**Table 1:** Relationships of the FA procedures to the CompFA model.

Proc.	Model	Note	Specific Factor and Errors	Estimate $\approx$ True	
LVFA	**x**$=$ ${\varvec{\Lambda }}$ **f** $+ {\varvec{\Psi }}$ **u**	${\varvec{\Psi }}\mathbf{u } ={\varvec{\Theta }}\mathbf{s }+ \mathbf{e }$	${\varvec{\Theta }}$ **s** & **e** unseparated	${\varvec{\Psi }}^{2} \approx$ $\underline{\varvec{\Theta }}^{2} +{\underline{{\mathbf{D}}}}_{\mathrm{EE}}$	${\varvec{\Lambda }}$ $\approx$ $\underline{\varvec{\Lambda }}$
MDFA	**X** $=$ **F**${\varvec{\Lambda }}^{\prime } +$ **S**${\varvec{\Theta }}$ $+$ **E**	$\downarrow$	diag($\mathbf{S }^\prime$ **E**) $={}_{p}$ **O**$_{p}$	${\varvec{\Theta }}^{2} \approx$ $\underline{{\varvec{\Theta }}}^{2} +{\underline{{\mathbf{D}}}}_{\mathrm{EE}}$	${\varvec{\Lambda }}$ $\approx$ $\underline{\varvec{\Lambda }}$
CDFA	**X** $=$ **F**${\varvec{\Lambda }}^{\prime } +$ **S**${\varvec{\Theta }}$ $+$ **E**	Nonrandom version	$\mathbf{S }^\prime$ **E** $= {}_{p}$ **O**$_{p}$	${\varvec{\Theta }}^{2} \approx$ $\underline{\varvec{\Theta }}^{2}$	${\varvec{\Lambda }}$ $\approx$ $\underline{\varvec{\Lambda }}$

The column furthest right in Table 1 shows how ${\varvec{\Psi }}^{2}$, ${\varvec{\Theta }}^{2}$, and $\varvec{\Lambda }$ are usually estimated in the FA procedures for the CompFA data matrix

17 $$\begin{aligned} {\mathbf{X}} ={\underline{{\mathbf{F}}}}\,{\varvec{\underline{\Lambda }}'} +{{{\underline{{\mathbf{S}}}}\,\underline{\varvec{\Theta }}}}+{\underline{{\mathbf{E}}}}\quad \hbox {with}\quad {\underline{{\mathbf{D}}}}_{\text {EE}} = {\text {diag}}({\underline{{\mathbf{C}}}}_{\mathrm{EE}} ) =\frac{1}{n} \text {diag}({\underline{{\mathbf{E}}}}^\prime {{\underline{{\mathbf{E}}}}}), \end{aligned}$$

i.e., the observations underlaid by (4) with **F**, $\varvec{\Lambda }$, **S**, $\varvec{\Theta }$, and **E** set to particular matrices ${{\underline{\mathbf{F}}}}$, $\underline{\Lambda }$, $\underline{\mathbf{S}}$, $\underline{{\varvec{\Theta }}}$, and ${\underline{{\mathbf{E}}}}$, respectively. Here, the latter matrices have been underlined for the sake of indicating that particular values are substituted into the elements of those matrices. The final (third) subsections in Sect. 2–4 are particularly concerned with how the true $\underline{\varvec{\Theta }}^{\, }$ and ${\underline{{\mathbf{D}}}}_{\mathrm{EE}}$ in (17) are related to LVFA, MDFA, and CDFA estimates, respectively, as outlined in the following two paragraphs.

In Sects. 2.3 and 3.3, we will discuss that the LVFA estimate of ${\varvec{\Psi }}^{2}$ approximates $\underline{\varvec{\Theta }}^{2}$ $+{\underline{{\mathbf{D}}}}_{\mathrm{EE}}$ and the MDFA one of $\varvec{\Theta }^{2}$ approximates $\underline{\varvec{\Theta }}^{2} +{{\underline{\mathbf{D}}}}_{\mathrm{EE}}$, respectively. The latter MDFA property of $\varvec{\Theta }^{2}$ $\approx$ $\underline{\varvec{\Theta }}^{2} +{\underline{\mathbf{D}}}_{\mathrm{EE}}$ is restated as $\varvec{\Theta }^{2}$ being contaminated by ${\underline{{\mathbf{D}}}}_{\mathrm{EE}}$. In Sect. 4.3, CDFA will be shown to provide the estimate $\varvec{\Theta }^{2\, }\approx$ $\underline{\varvec{\Theta }}^{2}$ differently from MDFA, with a discussion of how this difference follows from the CDFA solution satisfying (12), which is not met by the MDFA solution.

In Sects. 2.3, 3.3, and 4.3, we will also discuss that the uncorrelated error assumption (in Sect. 1.3) leads to the facts that are not covered in Table 1. The facts are summarized as follows. Two of the formulas with "$\approx$" in Table 1 are replaced by equalities only in strong uncorrelated error condition (15): $\varvec{\Psi }^{2} =$ $\underline{\varvec{\Theta }}^{2} +{{\underline{{\mathbf{D}}}}}_{\mathrm{EE}}$in LVFA and ${\varvec{\Theta }}^{2\, }=$ $\underline{\varvec{\Theta }}^{2}$ $+{{\underline{{\mathbf{D}}}}}_{\mathrm{EE}}$ in MDFA and CDFA, with all procedures fitting data perfectly. However, apart from that strong condition, the CDFA estimate of $\varvec{\Theta }^{2\, }$ can approximate ${\underline{{\varvec{\Theta }}}} ^{2}$, as described above.

The good recovery of loadings with ${\varvec{\Lambda }} \approx$ ${\underline{{\varvec{\Lambda }}}}$ in all procedures will be shown numerically in Sect. 5.

## Latent Variable Factor Analysis

In Sect. 2.1, we review the formulation of latent variable FA (LVFA) with the assumptions added to (1). Then, in Sect. 2.2, we show how those LVFA assumptions can follow from the CompFA ones. In Sect. 2.3, we discuss how the estimate of ${\varvec{\Psi }}^{2}$ approximates ${\underline{\varvec{\Theta }}^{2}} +{\underline{{\mathbf{D}}}}_{\mathrm{EE}}$, as shown in Table 1.

### Formulation

LVFA is modeled as (1), i.e., $\mathbf{x }={\varvec{\Lambda }} \mathbf{f } +{{\tilde{{\mathbf{e}}}}}={\varvec{\Lambda }} \mathbf{f } + \varvec{\Psi } \mathbf{u }$, where the expectations and covariances for **f** and ${{\tilde{\mathbf{e}}}}=\varvec{\Psi }\mathbf{u }$ are assumed to satisfy

18 $$\begin{aligned} E[\mathbf{f }] = \mathbf{0 }_{m},\quad C[\mathbf{f },\mathbf{f }] = \mathbf{I }_{m} , \end{aligned}$$

19 $$\begin{aligned} E[ \varvec{\Psi } \mathbf{u }] = \mathbf{0 }_{p},\quad C[\mathbf{f },\varvec{\Psi } \mathbf{u }] = {}_{m}\mathbf{O }_{p} , \end{aligned}$$

20 $$\begin{aligned} C[\varvec{\Psi } \mathbf{u }, {\varvec{\Psi }} \mathbf{u }] = {\varvec{\Psi }}^{2} . \end{aligned}$$

(e.g., Bartholomew, Knott, & Moustaki, 2011; Yanai & Ichikawa, 2007). Here, the diagonal elements of ${\varvec{\Psi }}^{2\, }$ are called unique variances, as they stand for the variances of the unique factor part ${\varvec{\Psi }}\mathbf{u }$.

LVFA assumptions (18)–(20) imply that inter-variable covariance matrix *C*[**x**, **x**] for (1) is expressed as

21 $$\begin{aligned} C[\mathbf{x }, \mathbf{x }] = C[ {\varvec{\Lambda }} \mathbf{f } + {\varvec{\Psi }} \mathbf{u }, {\varvec{\Lambda }} \mathbf{f } + {\varvec{\Psi }} \mathbf{u }] = {\varvec{\Lambda }}{\varvec{\Lambda }}^{\prime } + {\varvec{\Psi }}^{2}. \end{aligned}$$

Thus, ${\varvec{\Lambda }}$ and ${\varvec{\Psi }}^{2}$ can be estimated so that model-based (21) approximates its data-based counterpart **C**$_\mathrm{XX} = n^{-1} \mathbf{X }^\prime \mathbf{X }$. This estimation can be attained by minimizing

22 $$\begin{aligned} f^{\hbox {LS}}({\varvec{\Lambda }} , {\varvec{\Psi }} ) = \Vert \mathbf{C }_\mathrm{XX\, }- C[\mathbf{x }, \mathbf{x }]\Vert ^{2\, }= \Vert \mathbf{C }_\mathrm{XX\, }- ( \varvec{\Lambda } \varvec{\Lambda }^{\prime } + {\varvec{\Psi }}^{2})\Vert ^{2} \end{aligned}$$

over $\varvec{\Lambda }$ and $\varvec{\Psi }^{2}$ (Harman & Jones, 1966), with $\Vert \mathbf{M }\Vert$ $^{2} =$ tr**M**$^\prime$ **M** denoting the squared Frobenius norm of a matrix **M**. Another estimation procedure is to minimize the function

23 $$\begin{aligned} f_\mathrm{{NL}}( \varvec{\Lambda }, \varvec{\Psi } )&= {\hbox {tr}}\mathbf{C }_{\mathrm{XX}}C[\mathbf{x }, \mathbf{x }]^{-1} - {\hbox {log}}| \mathbf{C }_\mathrm{{XX}}C[\mathbf{x }, \mathbf{x }]^{-1}| - p \nonumber \\&= \hbox {tr}\mathbf{C }_\mathrm{{XX}}( {\varvec{\Lambda }} {\varvec{\Lambda }}^{\prime } + {\varvec{\Psi }}^{2})^{-1} - {\hbox {log}}| \mathbf{C }_\mathrm{{XX}}( {\varvec{\Lambda }} {\varvec{\Lambda }}^{\prime } + {\varvec{\Psi }}^{2})^{-1}| - p , \end{aligned}$$

which is the negative of the log likelihood following from (21) and the additional normality assumptions for **f** and **u** (e.g., Bartholomew, Knott, & Moustaki, 2011; Yanai & Ichikawa, 2007).

### Relationship to the CompFA Model

LVFA model (1) and assumptions (18)–(20) do not include the specific factor and error parts, which differs from CompFA model (3) and its assumptions. However, (1) is linked to (3) through (2). Further, it is shown next how LVFA constraints (18)–(20) follow from the CompFA counterparts:

#### Theorem 2

Under (1)–(3), LVFA assumptions (18)–(20) follow from CompFA ones (5)–(7) and uncorrelated error assumption (14).

#### Proof

Obviously, (18) follows, since its two equations appear in (5) and (6). Next, $E[\varvec{\Psi }\mathbf{u }]= \mathbf{0 }_{p}$ and *C*[**f**, $\varvec{\Psi }\mathbf{u }] =_{\,m}$ **O**$_{p}$ in (19) follow from (5)–(7) under (2), since (2) and (5) imply $E[{\varvec{\Psi }}\mathbf{u }]={\varvec{\Theta }} E[\mathbf{s }]+ E[\mathbf{e }] =\mathbf{0 }_{p}$, while (2), (6), and (7) lead to $C[\mathbf{f },\varvec{\Psi }\mathbf{u }] = C[\mathbf{f }, {\varvec{\Theta }} \mathbf{s} +\mathbf{e }] = C[\mathbf{f }, \mathbf{s }] {\varvec{\Theta }}^{\prime } + C[\mathbf{f },\mathbf{e }] =_{\,m}$ **O**$_{p}$. The remaining task is to show how (20) follows. Its left side is rewritten, using (2), (6), and (7), as $C[{\varvec{\Psi }} \mathbf{u }, {\varvec{\Psi }} \mathbf{u }] = C[ {\varvec{\Theta }} \mathbf{s }+ \mathbf{e }, {\varvec{\Theta }} \mathbf{s }+ \mathbf{e }] ={\varvec{\Theta }}^{2} + C[\mathbf{e\mathrm{,}e }]$. For this to equal the right side of (20), i.e., nonnegative diagonal ${\varvec{\Psi }}^{2}$, *C*[**e**, **e**] must equal ${\varvec{\Psi }}^{2}$ – ${\varvec{\Theta }}^{2}$ which is also nonnegative diagonal. This holds true for (14), i.e., ${\varvec{\Psi }}^{2}$ – ${\varvec{\Theta }}^{2} = D[\mathbf{e,e }]$. $\square$

This theorem shows that the LVFA assumptions follow from adding uncorrelated error assumption (14) to the standard CompFA ones in Sect. 1.1. However, LVFA can be performed for CompFA data (17), independently of whether its errors satisfy (14) or not. In the next subsection, how LVFA behaves for data (17) is discussed.

### Behaviors for CompFA Data

CompFA data matrix (17) leads to the covariance matrix

24 $$\begin{aligned} \mathbf{C }_{\mathrm{XX}} ={{\underline{{\varvec{\Lambda }}}\,{\underline{{\varvec{{\Lambda }}}}}'}}+ \underline{\varvec{\Theta }}^{2\, }+{{\underline{\mathbf{C}}}}_{\mathrm{EE}} , \end{aligned}$$

i.e., (13) with $\varvec{\Lambda }$, $\varvec{\Theta }^{2}$, and **C**$_{\mathrm{EE}}$ set to particular matrices $\underline{\varvec{\Lambda }}$, $\underline{\varvec{\Theta }}^{2}$, and ${{\underline{\mathbf{C}}}}_{\mathrm{EE}} =n^{-1}{{\mathbf{{E}}'{\mathbf{E}}}}$, respectively. Let us consider the LVFA solution for (24).

Loss functions (22) and (23) are known to be minimized for

25 $$\begin{aligned} \varvec{\Psi }^{2} = \hbox {diag}(\mathbf{C }_\mathrm{XX\, }- {\varvec{\Lambda }}{\varvec{ \Lambda }}^{\prime } ) \end{aligned}$$

for a given $\varvec{\Lambda }$ (e.g., Mulaik, 2010, (8.47), (8.80)). Using (24) in (25), this can be rewritten as $\varvec{\Psi }^{2\, }=$ diag(${{\underline{{\varvec{\Lambda }}}\,\underline{{\varvec{{{\Lambda }}}}}'}}+{{\underline{{\varvec{\Theta }}}}}^{2}+{{\underline{\mathbf{C}}}}_{\mathrm{EE}} -$ ${\varvec{\Lambda }}{\varvec{\Lambda }}^{\prime } ) =$ diag(${{\underline{{\varvec{\Lambda }}}\,\underline{\varvec{{{{\Lambda }}}}}'}}- {\varvec{\Lambda }} {\varvec{\Lambda }}^{\prime } ) +{\underline{{\varvec{{{\Theta }}}}}}^{2}+{\underline{{\mathbf{D}}}}_{\mathrm{EE}}$. This implies

26 $$\begin{aligned} \varvec{\Psi }^{2} \approx {\underline{{\varvec{{{\Theta }}}}}}^{2}+{\underline{{\mathbf{D}}}}_{\mathrm{EE}} \end{aligned}$$

for $\varvec{\Lambda }\varvec{\Lambda }^{\prime } \approx {{\underline{{\varvec{\Lambda }}}\,\underline{{{\varvec{{\Lambda }}}}}'}}$; if estimated $\varvec{\Lambda }\varvec{ \Lambda }^{\prime }$ approximates the true counterpart ${{\underline{{\varvec{\Lambda }}}\,\underline{{\varvec{{{\Lambda }}}}}'}}$, each of the diagonal elements in $\varvec{\Psi } ^{2}$, i.e., a unique variance, can be the estimate of the sum of the corresponding true specific and error variances, though these two cannot be estimated separately.

The next corollary, which follows from Theorem 1, shows that formula (26) with "$\approx$" is replaced by the equality $\varvec{\Psi }^{2}$ $={{\underline{{\varvec{{\Theta }}}}}}^{2}+{{\underline{\mathbf{D}}}}_{\mathrm{EE}}$, if (24) satisfies strong uncorrelated error condition (15):

#### Corollary 1

For (15), (24) is restricted to **C**$_{\mathrm{XX}} ={{\underline{{\varvec{\Lambda }}}\,{\underline{{\varvec{{\Lambda }}}}}'}}+{{\underline{{\varvec{{\Theta }}}}}}^{2}+{\underline{{\mathbf{D}}}}_{\mathrm{EE}}$. For this matrix, LVFA loss functions (22) and (23) can attain their lower limit zero with the solution of $\varvec{\Psi }^{2}$ given by $\varvec{\Psi }^{2\, }={{\underline{{\varvec{{\Theta }}}}}}^{2}+{{\underline{\mathbf{D}}}}_{\mathrm{EE}}$ and that of $\varvec{\Lambda }$ satisfying $\varvec{\Lambda } \varvec{\Lambda }^{\prime } ={\underline{{\varvec{\Lambda }}}\,\underline{\varvec{\Lambda }}'}$.

#### Proof

The matrix difference **C**$_{\mathrm{XX}} -$ ($\varvec{\Lambda } \varvec{\Lambda }^{\prime } +$ $\varvec{\Psi }^{2})$ in (22) and the product **C**$_{\mathrm{XX}}$($\varvec{\Lambda } \varvec{\Lambda }^{\prime } +$ ${\varvec{\Psi }}^{2})^{-1}$ in (23) are rewritten, using **C**$_\mathrm{{XX}} ={{\underline{{\varvec{\Lambda }}}\,{\underline{{\varvec{{\Lambda }}}}}'}}+{\underline{\varvec{\Theta }}}^{2}+{{\underline{\mathbf{D}}}}_{\mathrm{EE}}$, as ${{\underline{{\varvec{\Lambda }}}\,{\underline{{\varvec{{\Lambda }}}}}'}}+{{\underline{{\varvec{{\Theta }}}}}}^{2}+{{\underline{\mathbf{D}}}}_{\mathrm{EE}} -$ ($\varvec{\Lambda }\varvec{\Lambda }^{\prime } + {\varvec{\Psi }}^{2})$ and (${{\underline{{\varvec{\Lambda }}}\,{\underline{{\varvec{{\Lambda }}}}}'}}+{{\underline{{\varvec{{\Theta }}}}}}^{2}+{{\underline{\mathbf{D}}}}_{\mathrm{EE}} )$( $\varvec{\Lambda } \varvec{\Lambda }^{\prime } +\varvec{\Psi }^{2})^{-1}$, respectively. The former difference can be $_{p}$ **O**$_{p}$, and the latter product can be **I**$_{p}$, which allows (22) and (23) to attain zeros, for $\varvec{\Lambda } \varvec{\Lambda }^{\prime } ={{\underline{{\varvec{\Lambda }}}\,{\underline{{\varvec{{\Lambda }}}}}'}}$ and $\varvec{\Psi }^{2} = {{\underline{{\varvec{{\Theta }}}}}}^{2}+{\underline{{\mathbf{D}}}}_{\mathrm{EE}}$. $\square$

The result in this corollary is referred to in Sect. 3.3.

## Matrix Decomposition Factor Analysis

In Sect. 3.1, we review the original formulation of matrix decomposition FA (MDFA). Then, in Sects. 3.2 and 3.3, we discuss two properties of the MDFA solution shown in Table 1. One property is the solution’s satisfying diag($\mathbf{S }^\prime$ **E**) $=_{\,p}$ **O**$_{p}$ but not (12), which implies that MDFA can be reformulated with constraints that are less restrictive than the CompFA assumptions, as discussed in Sect. 3.2. The other property $\varvec{\Theta }^{2}$ $\approx$ $\underline{\varvec{\Theta }}^{2} +{\underline{{\mathbf{D}}}}_{\mathrm{EE}}$ is suggested by a theorem to be presented in Sect. 3.3.

### Original Formulation

For the data matrix **X** with **1**$_{n}^\prime$ **X** $=$ **0**$_{p}^\prime$, MDFA is formulated as minimizing the least squares function for (4), i.e.,

27 $$\begin{aligned} f({\mathbf{F}}, \varvec{\Lambda } , \mathbf{S }, \varvec{\Theta } ) =\frac{1}{n}\Vert \mathbf{E }\Vert ^{2} =\frac{1}{n}\Vert {\mathbf{X}} - {\mathbf{F}}\varvec{\Lambda }^{\prime } - \mathbf{S }\varvec{\Theta } \Vert ^{2} , \end{aligned}$$

over **F**, $\varvec{\Lambda }$, **S**, and $\varvec{\Theta }$ subjec to constraints (9) and (10). In the MDFA literature (e.g., Adachi & Trendafilov, 2018; Sočan, 2003; Stegeman, 2016), **1**$_{n}{}^\prime$ **E** $=$ **0**$_{p}{}^\prime$ in (9) has not been described, but this is trivial, since **1**$_{n}{}^\prime$ **E** $=$ **0**$_{p}{}^\prime$ follows from the other equations in (9), (4), and **1**$_{n}{}^\prime$ **X** $=$ **0**$_{p}{}^\prime$.

One difference in (27) from the LVFA loss functions, besides the former being based on (4), is that (22) and (23) in LVFA do not include the factor scores, while (27) includes those scores as **F** and **S** to be estimated. However, infinite solutions of **F** and **S** exist that minimize (27); the optimal **F** and **S** are not unique (Adachi & Trendafilov, 2018, Sect. 4). Thus, only the solutions of $\varvec{\Lambda }$ and $\varvec{\Theta }$ are interpreted in MDFA, as are those of $\varvec{\Lambda }$ and $\varvec{\Psi }$ in LVFA.

Though (27) is defined using data matrix **X**, the MDFA solution of $\varvec{\Lambda }$ and $\varvec{\Theta }$ can be obtained only if the covariance matrix **C**$_{{\mathrm{XX}}} = n^{-1}\mathbf{X }^\prime$ **X** is available, i.e., even if the original **X** is unavailable (Adachi, 2012, 2020). This can be captured in the fact that (27) can be expanded as $n^{-1}\hbox {tr}(\mathbf{X }^\prime \mathbf{X } + \varvec{\Lambda }^{\prime } \mathbf{F }^\prime \mathbf{F }\varvec{\Lambda } + \varvec{\Theta } \mathbf{S }^\prime \mathbf{S }\varvec{\Theta }) - 2n^{-1}\hbox {tr}(\mathbf{X }^\prime \mathbf{F }\varvec{\Lambda }^{\prime } +\mathbf{X }^\prime \mathbf{S }\varvec{\Theta } -\varvec{\Lambda } \mathbf{F }^\prime \mathbf{S }\varvec{\Theta })$ and simplified using (10) as tr**C**$_{\mathrm{XX}} + \hbox {tr}{\varvec{\Lambda }}{\varvec{\Lambda }}^{\prime } +$ tr$\varvec{\Theta }^{2} -$ 2tr**C**$_{{\mathrm{XF}}} \varvec{\Lambda }^{\prime } -$ 2tr**C**$_{{\mathrm{XS}}} \varvec{\Theta }$, in which **X** does not appear. Here, **C**$_{\mathrm{XF}} = n^{-1}\mathbf{X }^\prime$ **F** and $\mathbf{C }_{\mathrm{XS}} = n^{-1}\mathbf{X }^\prime \mathbf{S }$ contain the covariances of variables to factors and are uniquely determined for given $\varvec{\Lambda }$ and $\varvec{\Theta }$ (Adachi & Trendafilov, 2018, p. 411). On the other hand, the optimal $\varvec{\Lambda }$ and $\varvec{\Theta }$ can be obtained for given **C**$_{\mathrm{XF}}$ and **C**$_{\mathrm{XS}}$(Adachi & Trendafilov, 2018, p. 410). Thus, the optimal updates of the block matrices [**C**$_{\mathrm{XF}}$,**C**$_{\mathrm{XS}}$] and [$\varvec{\Lambda }$, $\varvec{\Theta }$] are iterated alternately to provide the solution of [$\varvec{\Lambda }$, $\varvec{\Theta }$] in Adachi’s (2012, 2020) MDFA algorithm.

### Reformulation from Properties of the Solution

Besides (9) and (10) imposed as constraints in MDFA, (11) and (12) are included in the standard CompFA assumptions (Sect. 1.2). Thus, we must note whether (11) and (12) are satisfied by the MDFA solution, i.e., the parameter estimates and **E** resulting in the minimization of (27) under (9) and (10). Adachi and Trendafilov (2018, Theorem 4.1) show that the solution satisfies (11) and the diagonal part of (12), i.e.,

28 $$\begin{aligned} \hbox {diag}(\mathbf{S }^\prime \mathbf{E }) ={} _{p}\mathbf{O }_{p} , \end{aligned}$$

but does not meet the off-diagonal part of (12) with **S**$^\prime$ **E**– diag(**S**$^\prime$ **E**) $\ne _{\, p}$ **O**$_{p}$ in general. That is, the MDFA solution satisfies standard CompFA assumptions (9)–(11) but does not meet (12).

The MDFA solution satisfying (11) and (28) implies that these equations can be included in the constraints. That is, MDFA can be reformulated as minimizing (27) over **F**, $\varvec{\Lambda }$, **S**, and $\varvec{\Theta }$ subject to constraints (9)–(11) and (28). Here, (28) being only the diagonal part of (12) implies that the MDFA formulation is less restrictive than the CompFA model, in that (12) is relaxed as (28). In contrast, the theorems presented in Sect. 4.2 show that the CDFA solutions satisfy CompFA constraints (9)–(12) completely.

### Behaviors for CompFA Data

In this section, we consider how MDFA behaves for CompFA data matrix (17). The next corollary, which follows from Theorem 1, shows the MDFA solution in strong uncorrelated error condition (15).

#### Corollary 2

For data matrix (17) satisfying ${{\underline{\mathbf{C}}}}_{\mathrm{EE}} ={{{\underline{\mathbf{D}}}}}_{\mathrm{EE}}$, i.e., (15) given by setting **E** to ${\underline{\mathbf{E}}}$, MDFA loss function (27) can attain its lower limit zero for the solution satisfying **F**$\varvec{\Lambda }^\prime ={{\underline{\mathbf{F}}\,{\underline{{\varvec{{\Lambda }}}}}'}}$, $\varvec{\Theta }$ $=\underline{{\varvec{\tilde{\Theta }}}}$, **S** $={{\underline{{\tilde{{\mathbf{S}}}}}}}$ with $\underline{{\varvec{\tilde{{{\Theta }}}}}}=({\underline{{\varvec{\Theta }}}}^{2}+{{{\underline{\mathbf{D}}}}}_{\mathrm{EE}} )^{1/2}$ and ${{\underline{{\tilde{\mathbf{S}}}}}}=({{\underline{\mathbf{S}}\,\underline{{\varvec{{\Theta }}}}}}+{{{\underline{\mathbf{E}}}}}){\underline{{\tilde{{\varvec{\Theta }}}}}}^{-1}$.

#### Proof

Theorem 1 shows that (17) can be rewritten into the error-free form **X** $={{\underline{\mathbf{F}}\,\underline{{{\varvec{\Lambda } }}}'}}+{{\underline{{\tilde{{\mathbf{S}}}}}\,{\underline{\tilde{\varvec{\Theta }}}}}}$ for ${{\underline{\mathbf{C}}}}_{\mathrm{EE}} ={\underline{{\mathbf{D}}}}_{\mathrm{EE}}$. Substituting **X** $={{\underline{\mathbf{F}}\,{\underline{{\varvec{{\Lambda }}}}}'}}+{\underline{\tilde{\mathbf{S}}}}\,{\underline{\tilde{\varvec{\Theta }}}}$ in (27), it is rewritten as $n^{-1}\left\| {{{\underline{\mathbf{F}}\,{\underline{{\varvec{{\Lambda }}}}}'}}+{{\underline{{\tilde{{\mathbf{S}}}}}\,\underline{\tilde{\varvec{\Theta }}}}}-{{{\mathbf{F}}{\varvec{\Lambda } }'}}-{{{\mathbf{S}}{\varvec{\Theta }}}}} \right\| ^{2}$. This can attain the lower limit zero for **F**$\varvec{\Lambda }^{\prime } ={{\underline{\mathbf{F}}\,{\underline{{\varvec{{\Lambda }}}}}'}}$, **S** $={{{\tilde{{\mathbf{S}}}}}}$, and $\varvec{\Theta }$ $={\underline{{\tilde{{{{\varvec{\Theta }}}}}}}}=(\underline{{\varvec{{\Theta }}}}^{2}+\underline{{\mathbf{D}}}_{\mathrm{EE}} )^{1/2}$. $\square$

By comparing Corollaries 1 and 2, we can find that both LVFA and MDFA perfectly fit the CompFA data with strong condition (15), then $\varvec{\Psi }^{2} = {{\underline{{\varvec{{\Theta }}}}}}^{2}+{{\underline{\mathbf{D}}}}_{\mathrm{EE}}$ in LVFA, but $\varvec{\Theta }^{2} ={{\underline{{\varvec{{\Theta }}}}}}^{2}+{\underline{{\mathbf{D}}}}_{\mathrm{EE}}$ in MDFA; its estimates of the specific variances are not their true values, and they are contaminated by the error variances in ${\underline{{\mathbf{D}}}}_{\mathrm{EE}}$. This shows an undesirable property of MDFA.

Though the data with (15) are unusual, as mentioned in Sect. 1.3, whether the above contamination can occur for usual CompFA data (17) that are not restricted by (15) is to be considered. For this consideration, we reparameterize the error matrix in (17) as

29 $$\begin{aligned} {{\underline{\mathbf{E}}}}= {{\underline{\mathbf{G}}{\underline{{\varvec{{\Gamma }}}}}'}} \quad \,\hbox {with}\,\quad \frac{1}{n}{{{\underline{\mathbf{G}}'}\,\underline{{\mathbf{G}}}}}= \mathbf{I }_{p\, }, \end{aligned}$$

and $\underline{\varvec{\Gamma }}$ being *p* $\times$ *p*. The next theorem suggests that the MDFA estimate of $\varvec{\Theta }^{2}$ can be contaminated by ${{\underline{\mathbf{D}}}}_{\mathrm{EE}}$, i.e., $\varvec{\Theta } ^{2}$ can be close to $\underline{\varvec{\Theta }}^{2\, }+{\underline{{\mathbf{D}}}}_{\mathrm{EE}}$:

#### Theorem 3

Consider the case where MDFA is performed for the data matrix specified as (17) with (29) and ${\mathrm{rank}}({\underline{{\mathbf{E}}}})={\mathrm{rank}}({{\underline{{\varvec{{\Theta }}}}}})=$ p. Let two matrices be defined as

30 $$\begin{aligned} {{\bar{\varvec{\Theta }}}}= (\underline{{{\varvec{\Theta }}}}^{2\,}+ \varvec{\Delta }^{2})^{1/2}\, \quad {\mathrm{and}}\, \quad {{\bar{\mathbf{S}}}}= ({{\underline{\mathbf{S}}\,\underline{{\varvec{{\Theta }}}}}}+{{\underline{\mathbf{G}}\varvec{\Delta } }}){{\bar{\varvec{\Theta }}}}^{-1}, \end{aligned}$$

with $\varvec{\Delta }$ $a\, p$ $\times$ p diagonal matrix. Then, the matrices ${{\bar{\varvec{\Theta }}}}$ and ${{\bar{\mathbf{S}}}}$ in (30) can be substituted into $\varvec{\Theta }$ and **S** in function (27), respectively, and ${{\bar{\mathbf{S}}}}$ can be substituted into **S** in (9) and (10). Moreover, if $\varvec{\Delta } =$ diag$(\underline{\varvec{\Gamma }})$ and $\mathbf{F}$ satisfies ${{\mathbf{F}'\bar{{\mathbf{S}}}}}=$ ${\mathbf{F}}^\prime {{\underline{\mathbf{E}}}}=\,_{m}{\mathbf{O}}_{p}$, then ${{\bar{\mathbf{S}}}}$ in (30) can be substituted into **S** in (28), and we can use $\varvec{\Theta } ={{\bar{\varvec{\Theta }}}} ,{\mathbf{S}} ={{\bar{\mathbf{S}}}}$, and (17) in (27) to rewrite this function as

31 $$\begin{aligned} f({\mathbf{F}}, {\varvec{\Lambda }} , {{\bar{\mathbf{S}}}}, {{\bar{\varvec{\Theta }}}}) =\frac{1}{n}\Vert {{\underline{\mathbf{F}}\,{\underline{{\varvec{\Lambda }}}}'}} -{\mathbf{F}}\varvec{\Lambda }^{\prime } \Vert ^{2} + {\mathrm{tr}}{\underline{{\mathbf{D}}}}_{\mathrm{EE}} -{\mathrm{tr}} {\varvec{\Delta }}^{2}. \end{aligned}$$

#### Proof

See Appendix 2.

Theorem 3 suggests that the MDFA estimate of $\varvec{\Theta }^{2}$ can be contaminated by ${\underline{{\mathbf{D}}}}_{\mathrm{EE}}$ with

32 $$\begin{aligned} \varvec{\Theta }^{2} \approx \underline{\varvec{\Theta }}^{2\, }+{\underline{{\mathbf{D}}}}_{\mathrm{EE}} , \end{aligned}$$

as explained next. The theorem allows us to consider that ${{\bar{\varvec{\Theta }}}}$ and ${{\bar{\mathbf{S}}}}$ in (30) can be the MDFA estimates of $\varvec{\Theta }$ and **S**, respectively. By substituting those ${{\bar{\varvec{\Theta }}}}$ and ${{\bar{\mathbf{S}}}}$ into $\varvec{\Theta }$ and **S** in the loss function (27) to be minimized, it can be rewritten as (31). This value can be small for **F**$\varvec{\Lambda }^{\prime } \approx {{\underline{\mathbf{F}}\,{\underline{{\varvec{{\Lambda }}}}}'}}$ and $\varvec{\Delta }^{2\, }\approx {\underline{{\mathbf{D}}}}_{\mathrm{EE}}$. This use in (30) and ${\varvec{\Theta }}$ $={{\bar{\varvec{\Theta }}}}$ lead to (32). It will be confirmed in Sect. 5 that (32) actually arises.

## Completely Decomposed Factor Analysis

In Sect. 4.1, we review Stegeman’s (2016) restrictive variant of MDFA that has been rephrased as completely decomposed FA (CDFA) through the theorems present in Sect. 4.2. They also allow CDFA to be reformulated so that it is perfectly matched with the CompFA model. In Sect. 4.3, we argue how the CDFA estimate of ${\varvec{\Theta }}^{2}$ approximates $\underline{{\varvec{\Theta }}}^{2}$ as shown in Table 1.

### Stegeman’s Factor Analysis Procedure

Stegeman’s (2016) original formulation of CDFA is to add the constraint

33 $$\begin{aligned} \mathbf{S }^\prime ({\mathbf{X}}-\mathbf{S }{\varvec{\Theta }} ) =_{\,p}\!\!\mathbf{O }_{p} \end{aligned}$$

to MDFA formulated as in Sect. 3.1: In CDFA, (27) is minimized over **F**, ${\varvec{\Lambda }}$, **S**, and ${\varvec{\Theta }}$ subject to (9), (10), and (33), for the data matrix **X** with **1**$_{n}^\prime$ **X** $=$ **0**$_{p}^\prime$.

As described in Stegeman (2016, p. 196), the CDFA solution can be obtained through the following three sequential steps: First, the optimal ${\varvec{\Theta }}$ subject to (33) can be obtained by performing ten Berge and Kiers’ (2001) minimum rank factor analysis (MRFA) for **C**$_\mathrm{XX}$ $= n^{-1}\mathbf{X }^\prime$ **X**. Then, the resulting ${\varvec{\Theta }}$ provides the solution of **S**. Finally, loss function (27), whose ${\varvec{\Theta }}$ and **S** are fixed to the ones resulting so far, is minimized over **F** and ${\varvec{\Lambda }}$ subject to (9) and (10). This minimization is attained for

34 $$\begin{aligned} {\mathbf{F}} =\sqrt{n} \mathbf{V }_{m} =\sqrt{n} ({\mathbf{X}}-{\mathbf{S}} {{{\varvec{{\varvec{\Theta }}}}}}){\mathbf{W}}_{m} {\varvec{\Omega }}_{m}^{-1}\,\, \hbox {and}\,\, {\varvec{\Lambda }} =\frac{1}{\sqrt{n} }{\mathbf{W}}_{m} {{\varvec{{\Omega }}}}_{m} \end{aligned}$$

through the singular value decomposition (SVD) of $\mathbf{X }-\mathbf{S }{\varvec{\Theta }}$ defined as

35 $$\begin{aligned} \mathbf{X } - \mathbf{S }{\varvec{\Theta }} = \mathbf{V }\varvec{\Omega } \mathbf{W }^\prime . \end{aligned}$$

Here, **V**$$^\prime $$ **V** $$=$$ **W**$$^\prime $$ **W** $$=$$ **I**$$_{q}$$, and $$\varvec{\Omega }$$ is the *q* $$\times $$ *q* diagonal matrix whose diagonal elements are arranged in decreasing order, with $$q =$$ rank(**X** − ** S**$${\varvec{\Theta }} )$$ and $$q \ge m$$ supposed. The matrices **V**$$_{m}$$ (*n* $$\times $$ *m*) and **W**$$_{m}$$ (*p* $$\times $$ *m*) in (34) contain the first *m* columns of **V** and **W**, respectively, with $$\varvec{\Omega }_{m\, }$$the upper-left $$m $$ $$\times $$ *m* diagonal block of $$\varvec{\Omega } $$.

The optimal **F** and ${\varvec{\Lambda }}$ in (34) are found to satisfy

36 $$\begin{aligned} \frac{1}{n}{{\mathbf{X'F}}}= {\varvec{\Lambda }} , \end{aligned}$$

since we can use (10), (34), (35), and $\mathbf{V }^\prime \mathbf{V }_{m}=[\mathbf{I }_{m}, _{m}\!\mathbf{O }_{q-m}]^\prime$ (from $\mathbf{V }^\prime \mathbf{V } = \mathbf{I }_{q})$ to derive (36) as

$$\begin{aligned} \frac{1}{n}{{\mathbf{X}'}}{\mathbf{F}}= \frac{1}{n}{{\mathbf{X}'}}{\mathbf{F}}-\frac{1}{n}{{\varvec{{{\Theta }}}}{\mathbf{S}}'}{\mathbf{F}}=\frac{1}{n}({\mathbf{X}}-{{{\mathbf{S}}{\varvec{\Theta }} }}{)}'{\mathbf{F}}=\frac{1}{n}{{{\mathbf{W}}\varvec{\Omega } \mathbf{V}'}}\left( {\sqrt{n} {\mathbf{V}}_{m} } \right) =\frac{1}{\sqrt{n} }{\mathbf{W}}_{m} {\varvec{\Omega }}_{m} = {\varvec{\Lambda }} . \end{aligned}$$

As in MDFA, the optimal **F** and **S** cannot be uniquely determined, but their estimation can be skipped to obtain the optimal ${\varvec{\Lambda }}$ and ${\varvec{\Theta }}$, only if the covariance matrix **C**$_\mathrm{{XX}}$ is available. This is shown using the fact that (10) and (33) lead to **X**$^\prime$ **S** $= n {\varvec{\Theta }}^{2}$. This, (33), and (35) imply that the SVD of (**X** − **S**${\varvec{\Theta }} )^\prime$(**X** − **S**${\varvec{\Theta }} )$ $=$ **X**$^\prime$ **X** $- n {\varvec{\Theta }}^{2} = n$(**C**$_\mathrm{{XX}}- {\varvec{\Theta }}^{2})$ can be defined as *n*(**C**$_\mathrm{{XX}} -$ ${\varvec{\Theta }}^{2}) =$ **W**$\varvec{\Omega }^{2}$ **W**$^\prime$ with ${\varvec{\Theta }}^{2}$ given by MRFA for **C**$_\mathrm{{XX}}$. This SVD can provide ${\varvec{\Lambda }}$ with (34).

### Reformulation Matched to the CompFA Model

The CDFA solution, i.e., the parameter estimates resulting in the minimization of (27) under (9), (10), and (33), satisfies (36). This implies that (36) can be included in the constraints: CDFA can also be formulated as minimizing (27) subject to (9), (10), (33), and (36). Here, constraints (33) and (36) are proved to be equivalent to (12) and (11), respectively, under (4) and (10), in the next two theorems.

#### Theorem 4

Under (4) and (10), (33) is equivalent to (12), *i*.*e*., **S**$^\prime$ **E** $={}_{\,m}$ **O**$_{p}$.

#### Proof

First, (33) $\rightarrow$ (12) is proved as follows: Using (4), we can rewrite (33) as **S**$^\prime$(**F**${\varvec{\Lambda }}^{\prime } +$ **E**) $=_{\,p}$ **O**$_{p}$, which implies (12), i.e., **S**$^\prime$ **E** $=_{\,m}$ **O**$_{p}$, from (10). Next, (12) $\rightarrow$ (33) is proved as follows: We can use (4) and (10) to rewrite (12) as **S**$^\prime (\mathbf{X }-\mathbf{F }$ ${\varvec{\Lambda }}^{\prime }-\mathbf{S} {\varvec{\Theta }} ) =_{\, }$ **S**$^\prime (\mathbf{X }-\mathbf{S }{\varvec{\Theta }} ) =_{\,p}$ **O**$_{p}$, i.e., (33). $\square$

#### Theorem 5

Under (4) and (10), (36) is equivalent to (11), *i*.*e*., **F**$^\prime$ **E** $=_{\,m}$**O**$_{\!p}$.

#### Proof

First, let us prove (36) $\rightarrow$ (11). The former is rewritten as **F**$^\prime$ **X**$=n {\varvec{\Lambda }}^{\prime }$. Using this, (4), and (10), we have **F**$^\prime$ **E**$=$ **F**$^\prime$(**X**–**F**${\varvec{\Lambda }}^{\prime } -$** S**${\varvec{\Theta }} ) = \mathbf{F }^\prime \mathbf{X }-\mathbf{F} ^\prime \mathbf{F }{\varvec{\Lambda }}^{\prime }=n {\varvec{\Lambda }}^\prime - n {\varvec{\Lambda }}^{\prime } = {}_{m}$ **O**$_{p}$, i.e., (11). Next, (11) $\rightarrow$ (36) is proved as follows: We can use (4) and (10) to rewrite (11) as **F**$^\prime (\mathbf{X }-\mathbf{F }{\varvec{\Lambda }}^{\prime } -\mathbf{S} {\varvec{\Theta }}) = \mathbf{F }^\prime \mathbf{X }-\mathbf{F} ^\prime \mathbf{F }{\varvec{\Lambda }}^{\prime }=\mathbf{F }^\prime \mathbf{X }- n {\varvec{\Lambda }}^{\prime } =_{\,m}\!\!\mathbf{O }_{p}$, which leads to (36). $\square$

These theorems and (10) show that **F**$^\prime$ **S** $=_{\,m}$ **O**$_{p}$, **F**$^\prime$ **E** $=_{\,m}$ **O**$_{p}$, and **S**$^\prime$ **E** $=_{\,p}$ **O**$_{p}$ in (10)–(12) hold true in the CDFA solution. These three equations imply that the common factors, specific factors, and errors are mutually decomposed completely, thus the name CDFA. This name is considered more suitable than another name, *constrained uniqueness FA*, used in Adachi (2019), as the CompFA model has not been considered in the latter naming. Further, the theorems show that Stegeman’s (2016) procedure in Sect. 4.1 can be reformulated as minimizing least squares function (27) for model (4) subject to its constraints (9)–(12). Thus, CDFA is perfectly matched to the CompFA model and its standard constraints in Sect. 2.2. Here, let us remember that MDFA can be reformulated as minimizing (27) subject to (9)–(11) and (28) (Sect. 3.2); this (28) is strengthened into (12) in CDFA.

### Behaviors for CompFA Data

In this section, we consider how CDFA behaves for CompFA data (17). At first, the following corollary shows the CDFA solution in strong uncorrelated error condition (15):

#### Corollary 3

CDFA can be substituted for MDFA in Corollary 2.

#### Proof

This is the same as the proof for Corollary 2, since (27) is also the CDFA loss function. $\square$

This corollary shows that the CDFA estimate of ${\varvec{\Theta }}^{2\, }$(specific variances) is contaminated by ${\underline{{\mathbf{D}}}}_{\mathrm{EE}}$(error variances) for the CompFA data with (15), as is the MDFA estimate. However, we can argue that the contamination is less likely to occur in CDFA than in MDFA for the usual CompFA data that are not restricted by (15). This argument follows from the fact that constraint (28), i.e., diag(**S**$^\prime$ **E**) $=_{\,p}$ **O**$_{p}$, in MDFA is strengthened as (12), i.e., **S**$^\prime$ **E** $=$ $_{p}$ **O**$_{p}$, in CDFA, as explained in the following paragraph.

In Sect. 3.3, we discussed that the MDFA estimate of $${\varvec{\Theta }} ^{2}$$ can be contaminated as (32), which follows from (30) with $${{\bar{\mathbf{S}}}}=$$ ($${{\underline{\mathbf{S}}\,\underline{{\varvec{{{\varvec{\Theta }} }}}}}}+{{\underline{\mathbf{G}}{\varvec{\Delta }} }}){{\bar{\varvec{{\varvec{\Theta }} }}}}^{-1}$$. This matrix and **E** lead to $${{{\bar{\mathbf{S}}}'}}{\mathbf{E}}=$$$$n{{\bar{\varvec{{{\Theta }}}}}}^{-1}$$ ($${\varvec{\Delta }}\underline{\varvec{\Gamma }}^{\prime }- {\varvec{\Delta }}^{2})$$ as shown by (A6) in Appendix 2. Here, $${\varvec{\Delta }} $$ is a *p* $$\times $$ *p* diagonal matrix and $$\underline{\varvec{\Gamma }}$$ is defined as in (29). In CDFA, the above equation for $${{{\bar{\mathbf{S}}}'}}{\mathbf{E}}$$ must be substituted into **S**$$^\prime $$ **E** in (12) as

37 $$\begin{aligned} {{{\bar{\mathbf{S}}}'{\mathbf{E}}}}= n{{\bar{\varvec{{\varvec{\Theta }}}}}}^{-1}( {\varvec{\Delta }} {\underline{\varvec{\Gamma }}}^{\prime } - {\varvec{\Delta }}^{2}) = {}_{p}\mathbf{O }_{p\, \, }, \end{aligned}$$

since (12) is included in the CDFA constraints as described in Sect. 4.2. The equivalence of (37) to strong uncorrelated error condition (15) is shown next:

#### Theorem 6

Substituting ${\underline{{\mathbf{E}}}}$ in (17) into **E** in (15), it is rewritten as ${{\underline{\mathbf{C}}}}_{\mathrm{EE}} ={\underline{{\mathbf{D}}}}_{\mathrm{EE}}$. This is equivalent to (37).

#### Proof

The last identity in (37) holds if and only if $\underline{\varvec{\Gamma }}$ is a diagonal matrix: $\underline{\varvec{\Gamma }}$ $=$ ${\varvec{\Delta }}$. This equivalence to ${{\underline{\mathbf{C}}}}_{\mathrm{EE}} ={\underline{{\mathbf{D}}}}_{\mathrm{EE}}$ is shown using (29) rewritten as ${{\underline{\mathbf{C}}}}_{\mathrm{EE}} =n^{-1}{{\mathbf{{E}}'{} \mathbf{E}}}=$ $\underline{\varvec{\Gamma }}$ $\underline{\varvec{\Gamma }}^{\prime }$. That is, $\underline{\varvec{\Gamma }}={\varvec{\Delta }}$ implies that ${{\underline{\mathbf{C}}}}_{\mathrm{EE}} =$ $\underline{\varvec{\Gamma }}$ $\underline{\varvec{\Gamma }}^\prime$ is also diagonal: ${{\underline{\mathbf{C}}}}_{\mathrm{EE}} ={\underline{{\mathbf{D}}}}_{\mathrm{EE}}$. On the other hand, ${{\underline{\mathbf{C}}}}_{\mathrm{EE}} ={\underline{{\mathbf{D}}}}_{\mathrm{EE}}$, i.e., ${{\underline{\mathbf{C}}}}_{\mathrm{EE}} =$ $\underline{\varvec{\Gamma }}$ $\underline{\varvec{\Gamma }}^{\prime }$ being diagonal, implies that $\underline{\varvec{\Gamma }}$ is diagonal: $\underline{\varvec{\Gamma }} ={\varvec{\Delta }}$. $\square$

This theorem shows that contamination (32) is less likely to occur in CDFA for the data that do not satisfy (15). On the other hand, even for such data, (32) can occur in MDFA, where **S**$^\prime$ **E** may not be $_{p}$ **O**$_{p}$, but only diag(**S**$^\prime$ **E**) $=$ $_{m}$ **O**$_{p}$ is required. These arguments are empirically supported in the next section.

## Simulation Study

We assess the performance of the FA procedures for the CompFA data in a simulation study. Its purposes and the data types to be simulated are detailed in Sect. 5.1. Data analysis and assessment procedures are described in Sect. 5.2, and the results are reported in Sects. 5.3–5.5.

### Purposes and Data Synthesis Procedures

In this study, the FA procedures are carried out for the CompFA data synthesized with the true loading matrix $\underline{{\varvec{\Lambda }}}$ and specific variance matrix $\underline{{\varvec{\Theta }}}^{2}$. The major purpose of this study is to numerically assess the following hypotheses:

$${[\hbox {H}_{1}]}$$
The CDFA estimate of ${\varvec{\Theta }}^{2}$ approximates the true $\underline{{\varvec{\Theta }}}^{2}$ better than the MDFA estimate.
$${[\hbox {H}_{2}]}$$
The MDFA estimate of ${\varvec{\Theta }}^{2}$approximates $\underline{{\varvec{\Theta }}}^{2}+{\underline{{\mathbf{D}}}}_{\mathrm{EE}}$, i.e., is contaminated by ${\underline{{\mathbf{D}}}}_{\mathrm{EE}}$, with ${\underline{{\mathbf{D}}}}_{\mathrm{EE}}$ the diagonal matrix including the true error variances.
$${[\hbox {H}_{3}]}$$
The LVFA estimate of unique variance matrix ${\varvec{\Psi }}^{2\, }$ approximates ${\underline{{\varvec{\Theta }}}^{2}} +{{\underline{{\mathbf{D}}}}_{\mathrm{EE}}}$.
$${[\hbox {H}_{4}]}$$
The estimates of ${\varvec{\Lambda }}$ in all procedures approximate the true $\underline{{\varvec{\Lambda }}}$.

Here, [H$_{1}$] follows from the discussion in Sect. 4.3, and hypotheses [H$_{2}$] and [H$_{3}$] are equivalent to (32) and (26), respectively. [H$_{4}$] has not been discussed, but rather has been presupposed for the discussions in Sects. 2–4. The estimates in the hypotheses are obtained, given the covariance matrix **C**$_\mathrm{{XX}} =$ $n^{-1}$ **X**$^\prime$**X** for the CompFA data. Thus, we describe below how the true $\underline{{\varvec{\Lambda }}}$ and $\underline{{\varvec{\Theta }}}^{2}$ are set and how they lead to **C**$_\mathrm{{XX}}$.

Let $U(\alpha$, $\beta )$ denote the uniform distribution for the interval [$\alpha$, $\beta$]. Each element of $\underline{{\varvec{\Lambda }}}$ and each diagonal one of $\underline{{\varvec{\Theta }}}^{2}$ are drawn from $U(-$1, 1) and *U*(0.1, 0.8), respectively, subject to rank($\underline{{\varvec{\Lambda }}} ) = m$. From the resulting $\underline{{\varvec{\Lambda }}}$ and $\underline{{\varvec{\Theta }}}^{2}$, we generate 12 types of **C**$_\mathrm{{XX}}$ with rank(**C**$_\mathrm{{XX}})$ constrained to be *p*. Here the 12 ($=$ 3 $\times$ 2 $\times$ 2) types are defined by combining the three levels of error correlations, two levels of error magnitudes, and two versions of the CompFA model, which are explained in the following paragraphs.

The two versions of the model correspond to its nonrandom (N) version (4) and random (R) version (3). The covariance matrix for the N version is given by (24) for (17) with ${{\underline{\mathbf{C}}}}_{\mathrm{EE}} =n^{-1}\sum \nolimits _{i=1}^n {{\mathbf{e}}_{i} {{\mathbf{e}'}}_{i} }$:

38 $$\begin{aligned} \mathbf{C }_\mathrm{{XX}} ={{\underline{{\varvec{\Lambda }}}\,\underline{{\varvec{{{\Lambda }}}}}'}}+ \underline{{\varvec{\Theta }}}^{2\, }+{{\underline{\mathbf{C}}}}_{\mathrm{EE}} ={{\underline{{\varvec{\Lambda }}}\,{\underline{{\varvec{{\Lambda }}}}}'}}+ \underline{{\varvec{\Theta }}}^{2\, }+\frac{1}{n}\sum \limits _{i=1}^n {{\mathbf{e}}_{i} {{\mathbf{e}'}}_{i}} . \end{aligned}$$

Here, error vectors **e**$_{i}$ ($i =$ 1, …, *n*) are chosen as **e**$_{i} = \alpha \varvec{\upvarepsilon }_{i}$, with $\varvec{\upvarepsilon }_{i}$ drawn from $N_{p}$(**0**$_{p}$, $\varvec{\Phi } )$, i.e., the *p*-variate normal distribution whose mean vector is **0**$_{p}$ and covariance matrix is $\varvec{\Phi } (p$ $\times$ *p*). How $\alpha$ and ${\varvec{\Phi }}$ are defined is described later. The matrix for the R version is given by

39 $$\begin{aligned} \mathbf{C }_\mathrm{{XX}}=\frac{1}{n}\sum \limits _{i=1}^n {{\mathbf{x}}_{i} {{\mathbf{x}'}}_{i} } =\frac{1}{n}\sum \limits _{i=1}^n {({{\underline{{\varvec{\Lambda }}}{\mathbf{f}}}}_{i} +{{\underline{{\varvec{{{{\Theta }}}}}}{\mathbf{s}}}}_{i} +{\mathbf{e}}_{i} )({{\underline{{\varvec{\Lambda }}}{\mathbf{f}}}}_{i} +{{\underline{{\varvec{{\Theta }}}}{\mathbf{s}}}}_{i} +{\mathbf{e}}_{i} {)}'} . \end{aligned}$$

This follows from (3), whose random vectors are followed by the observation-number subscript *i*. The factor score vectors in (39) are sampled with [**f**$$_{i}{}^\prime $$, **s**$$_{i}{}^\prime $$]$$\prime \sim $$ $$N_{m+p}$$(**0**$$_{m+p}$$, **I**$$_{m+p})$$.

The three levels of error correlations can be referred to as no, low, and high levels (C$$_{\mathrm{N}}$$, C$$_{\mathrm{L}}$$, and C$$_{\mathrm{H}})$$, while the two levels of error magnitudes can be called low and high levels (E$$_{\mathrm{L}}$$ and E$$_{\mathrm{H}})$$, respectively. Here, C, E, N, L, and H in the parentheses are abbreviations of correlation, error, no, low, and high, respectively. At the C$$_{\mathrm{N}}$$ level, $${\varvec{\Phi }}$$ is set to the diagonal matrix **D**$$_{\mathrm{R}}$$ whose diagonal elements are drawn from *U*(0.1, 0.8). At the C$$_{\mathrm{L}}$$ and C$$_{\mathrm{H}}$$ levels, $${\varvec{\Phi }}$$ is set to **D**$$_{\mathrm{R}}^{1/2}$$**RD**$$_{\mathrm{R}}^{1/2}$$, with **R** $$=$$ ($$r_{jk})$$ the *p* $$\times $$ *p* symmetric nonnegative-definite matrix whose elements are chosen as $$r_{jj} =$$ 1 and $$r_{jk} =\tau _{jk} \tilde{{r}}_{jk} $$ for $$j \ne k$$. Here, $$\tau _{jk\, }$$ is randomly set to 1 or $$-1$$, and $$\tilde{{r}}_{jk} $$ is drawn from $$N_{1}$$(0.2$$\rho $$, 0.05$$^{2}\rho ^{2})$$ subject to $$-1< \tilde{{r}}_{jk}< 1$$, with $$\rho = 1$$ for C$$_{\mathrm{L}}$$ and $$\rho = 2$$ for C$$_{\mathrm{H}}$$. The $$\alpha $$ value is set so that $$\hbox {tr}{\varvec{\Phi }} /\hbox {tr}{\varvec{\Lambda }} {\varvec{\Lambda }}^{\prime }=$$ 0.1 and 0.2 for the E$$_{\mathrm{L}}$$ and E$$_{\mathrm{H}}$$ levels, respectively. We should notice that $${\varvec{\Phi }}$$ is diagonal at the C$$_{\mathrm{N}}$$ level, but $${{\underline{\mathbf{C}}}}_{\mathrm{EE}} =n^{-1}\sum \nolimits _{i=1}^n {{\mathbf{e}}_{i} {{\mathbf{e}'}}_{i} } $$ obtained with $$\mathbf{e }_{i}\!\sim \!N_{p}(\mathbf{0 }_{p}, {\varvec{\Phi }})$$ is not necessarily diagonal; the C$$_\mathrm{N}$$ level does not exactly match (15). In Appendix 3, it is reported that a simulation study for **C**$$_{\mathrm{XX}}$$ satisfying (15) numerically demonstrated the facts in Corollaries 1–3.

The procedures in the last three paragraphs were replicated 500 times with the settings $n =$ 200, $p =$ 12, and $m =$ 3. Thus, we had 6000 ($=$12 types $\times$ 500 times) **C**$_{\mathrm{XX}}$.

### Data Analysis and Assessment Procedures

For each of the 6000 **C**$_{\mathrm{XX}}$, we performed MDFA, CDFA, and the two types of LVFA, i.e., the least squares LVFA (LS-LVFA) and maximum likelihood LVFA (ML-LVFA) for minimizing (22) and (23), respectively. Their algorithms are described in Appendix 4. In every procedure, ${\varvec{\Lambda }}$ has rotational indeterminacy; thus, it was rotated by the orthogonal Procrustes method (e.g., Adachi, 2020, p. 206), to optimally approximate the true counterpart ${\underline{{{\varvec{\Lambda }}}}}$ in a least squares sense. This approximation allows ${\varvec{\Lambda }}$ to be comparable to ${\underline{{{\varvec{\Lambda }}}}}$; thus, the Procrustes method has been typically used in previous simulation studies for FA (e.g., Adachi, 2013, Appendix D; Stegeman, 2016, Sect. 4.2).

The similarities between estimated parameters and their true values can be assessed with the smallness of a mean absolute difference (MAD). The MAD for loadings is defined as MAD($\underline{{\varvec{\Lambda }}}) =\Vert {\varvec{\Lambda }} -\underline{{\varvec{\Lambda }}}\Vert _{1}/(\textit{pm})$, where $\Vert {\varvec{\Lambda }} -\underline{{\varvec{\Lambda }}} \Vert _{1}$ denotes the $L_{1}$ norm of ${\varvec{\Lambda }} -$ $\underline{{\varvec{\Lambda }}}$, i.e., the sum of the absolute values of the elements in ${\varvec{\Lambda }} -\underline{{\varvec{\Lambda }}}$. The denominator *pm* in this definition is replaced by *p* in the following two MAD for *p* $\times$ *p* diagonal matrices:

40 $$\begin{aligned} \hbox {MAD}({{\underline{{\varvec{{\Theta }}}}}}^{2})= & {} \left\{ {{\begin{array}{ll} &{}{\frac{1}{p}\left\| {{{\varvec{{\Psi }}}}^{2}-{{\underline{{\varvec{{\Theta }}}}}}^{2}} \right\| _{1}} \hbox {for LVFA} \\ &{}{\frac{1}{p}\left\| {{{\varvec{{\Theta }}}}^{2}-{{\underline{{\varvec{{\Theta }}}}}}^{2}} \right\| _{1}} \hbox {for MDFA and CDFA} \\ \end{array} }\;} \right. , \end{aligned}$$

41 $$\begin{aligned} \hbox {MAD}({{\underline{{\varvec{{\Theta }}}}}}^{2}+{\underline{{\mathbf{D}}}}_{\mathrm{EE}} )= & {} \left\{ {{\begin{array}{ll} &{}{\frac{1}{p}\left\| {{{\varvec{{\Psi }}}}^{2}-({{\underline{{\varvec{{\Theta }}}}}}^{2}+{\underline{{\mathbf{D}}}}_{\mathrm{EE}} )} \right\| _{1}} \hbox {for LVFA} \\ &{}{\frac{1}{p}\left\| {{{\varvec{{\Theta }}}}^{2}-(\underline{{{\varvec{\Theta }}}}^{2}+{\underline{{\mathbf{D}}}}_{\mathrm{EE}} )} \right\| _{1}} \hbox {for MDFA and CDFA}\\ \end{array} }\;} \right. \end{aligned}$$

with ${\underline{{\mathbf{D}}}}_{\mathrm{EE}} ={\hbox {diag}}({{\underline{\mathbf{C}}}}_{\mathrm{EE}} )={\hbox {diag}}(n^{-1}\sum \nolimits _{i=1}^n {{\mathbf{e}}_{i} {{\mathbf{e}'}}_{i} } )$. Here, MAD in (40) and (41) for LVFA differs from those for MDFA and CDFA, as ${\varvec{\Psi }}^{2}$ corresponds to $\underline{\varvec{\Theta }}^{2}$ in LVFA. We obtain (41) in addition to (40), as both MAD are necessary for examining [H$_{2}$] and [H$_{3}$] in Sect. 5.1.

Let {${\varvec{\Lambda }}_\mathrm{{LS}}$, ${\varvec{\Psi }} _\mathrm{{LS}}$} and {${\varvec{\Lambda }} _{\mathrm{ML}}$, ${\varvec{\Psi }}_{\mathrm{ML}}$} denote the LS- and ML-LVFA estimates of {${\varvec{\Lambda }}$, ${\varvec{\Psi }}$}, respectively. The broad equivalence between {${\varvec{\Lambda }}_\mathrm{{LS}}$, ${\varvec{\Psi }} _\mathrm{{LS}}$} and {${\varvec{\Lambda }} _{\mathrm{ML}}$, ${\varvec{\Psi }}_{\mathrm{ML}}$} was found with the averages (standard deviations) of $\Vert {\varvec{\Lambda }} _\mathrm{{LS}} - {\varvec{\Lambda }}_{\mathrm{ML}}\Vert$ $_{1}$/(*pm*) and $\Vert {\varvec{\Psi }} _\mathrm{{LS}}^{2} - {\varvec{\Psi }}_{\mathrm{ML}}^{2}\Vert _{1}$/*p* over the solutions for 5935 ($= 6000 - 65$) **C**$_{\mathrm{XX}}$ being 0.010 (0.011) and 0.022 (0.019), respectively. Here, we have removed the 65 **C**$_{\mathrm{XX}}$ for which LS-LVFA provided improper solutions with negative unique variances. As described in Appendix 4, such improper solutions show an undesirable property of LS-LVFA. These results allowed us to only consider the ML-LVFA solutions; thus, we refer to only ML-LVFA as LVFA, hereafter.

Now, we have the MAD values for the three matrices (M), $\underline{{\varvec{\Lambda }}}$, $\underline{{\varvec{\Theta }}}^{2}$, and $\underline{{\varvec{\Theta }}}^{2}+{\underline{{\mathbf{D}}}}_{\mathrm{EE}}$, which were obtained from each solution of the three FA procedures (P) performed for the 6000 ($=$ 3 $\times$ 2 $\times$ 2 $\times$ 500) **C**$_{\mathrm{XX}}$. Here, these **C**$_{\mathrm{XX}}$ are classified by combining the three levels of error correlations (C), two levels of error magnitudes (E), two versions (V) of the model, and 500 replications (R). In order to assess the hypotheses in Sect. 5.1, we performed analysis of variance of the randomized block design (ANOVA-RBD) (e.g., Kirk, 2013) for the MAD values. Here, P, M, C, E, and V were treated as treatments and R was treated as a block factor; the factorial design can be expressed as P $\times$ M $\times$ C $\times$ E $\times$ V with R consisting of 500 blocks, with the sets of the levels in P, M, C, E, and V being {LVFA, MDFA, CDFA}, {$\underline{{\varvec{\Lambda }}}$, $\underline{{\varvec{\Theta }}}^{2}$,$\underline{{\varvec{\Theta }}}^{2}\!+{\underline{{\mathbf{D}}}}_{\mathrm{EE}}$}, {E$_{\mathrm{L}}$ E$_{\mathrm{H}}$}, and {N,R}, respectively.

The above ANOVA-RBD provided the *F* values for main and interaction effects in Table 2. We do not use those values for statistical hypothesis testing, since this is senseless for our simulated data, whose sample size is too large to reject null hypotheses. But rather, we consider the *F* values as standing for the sizes of the effects, as the number of the levels in each treatment is restricted to two or three; thus, it makes sense to compare the *F* values. We regard the effects with $F \ge$ 24,000 as substantial enough, though the lower limit of 24,000 is a benchmark threshold chosen because 24,000 is far greater than the largest one ($=$ 14,491.0) among the *F*-values less than 24,000. Thus, the five effects boldfaced in Table 2 are regarded as substantial.

**Table 2:** *F* values resulting in ANOVA-RBD with the substantial effects boldfaced whose *F* values > 24,000.

Effect	Source and *F* value							
Main	P	**M**		C	**E**	**V**		Block
	444.5	**195361.0**		14491.0	**107830.5**	**26992.8**		36.4
Two-way interaction	**P**${\,\times \,}$**M**	P${\,\times \,}$C		P${\,\times \,}$E	P${\,\times \,}$V	M${\,\times \,}$C		**M**${\,\times \,}$**E**
	**42997.4**	67.7		213.6	952.1	5845.3		**24629.0**
	M${\,\times \,}$V	C${\,\times \,}$E		C${\,\times \,}$V	E${\,\times \,}$V			
	8393.9	1938.9		235.3	341.9			
Three-way interaction	P${\,\times \,}$M${\,\times \,}$C	P${\,\times \,}$M${\,\times \,}$E		P${\,\times \,}$ M${\,\times \,}$V	P${\,\times \,}$C${\,\times \,}$E	P${\,\times \,}$C${\,\times \,}$V		P${\,\times \,}$E${\,\times \,}$V
	2051.2	4591.3		400.7	16.3	27.7		77.7
	M${\,\times \,}$C${\,\times \,}$E	M${\,\times \,}$ C${\,\times \,}$V		M${\,\times \,}$E${\,\times \,}$V	C${\,\times \,}$E$\times$ V			
	1090.1	908.0		102.3	4.8			
Higher-order interactions	P${\,\times \,}$ M${\,\times \,}$C${\,\times \,}$E		P${\,\times \,}$ M${\,\times \,}$C${\,\times \,}$V		P${\,\times \,}$ M${\,\times \,}$E${\,\times \,}$V		P${\,\times \,}$C${\,\times \,}$E${\,\times \,}$V	
	291.1		277.6		28.6		1.3	
	M${\,\times \,}$C${\,\times \,}$E${\,\times \,}$V		P${\,\times \,}$M${\,\times \,}$C${\,\times \,}$ E${\,\times \,}$V					
	32.2		7.8					

### Averages for Substantial Effects

The averages of MAD for the levels associated with the substantial effects are presented in Table 3. The averages for the main effect of V in Panel (C) show that the MAD for R version (39) is greater than that for N one (38); this can be attributable to the fact that (39) has higher randomness. The averages for the other main effects in the bottom rows of Panels (A) and (B) can be interpreted from the interactions considered next.

The averages for the P $\times$ M levels in Panel (A) show that [H$_{1}$]–[H$_{4}$] in Sect. 5.1 were supported: [H$_{1}$] is supported by the CDFA average 0.126 for (40) being less than the MDFA counterpart 0.217, [H$_{2}$] and [H$_{3}$] are supported by the result that the LVFA and MDFA averages for (41) are less than those for (40), and [H$_{4}$] is supported by the averages of MAD($\underline{{\varvec{\Lambda }}})$ being small and almost equivalent among the procedures.

Panel (B) shows that every MAD increases with the change from E$_{\mathrm{L}}$ to E$_{\mathrm{H}}$, but the increments in (40) and (41) values are much larger than those in MAD($\underline{{\varvec{\Lambda }}})$. This difference in the increments can be interpreted by taking into account the related three-way interaction, as explained in the next subsection.

**Table 3:** Averages of MAD for substantial effects with those for main effects shown by boldfaced italic letters.

(A) Procedure $\times$ Matrix				(B) Matrix $\times$ Error			(C) Version	
	$\underline{{\varvec{\Lambda }}}$	$\underline{{\varvec{\Theta }}}^{2}$	$\underline{{\varvec{\Theta }}}^{2}+ {\underline{{\mathbf{D}}}}_{\mathrm{EE}}$		E$_\mathrm{L}$	E$_\mathrm{H}$	***Nonrandom***	***0.099***
LVFA	0.045	0.228	0.074	$\underline{{\varvec{\Lambda }}}$	0.037	0.053	***Random***	***0.131***
MDFA	0.044	0.217	0.073	$\underline{{\varvec{\Theta }}}^{2}$	0.132	0.249		
CDFA	0.046	0.126	0.182	$\underline{{\varvec{\Theta }}}^{2}+$ $\underline{{\mathbf{D}}}_{\mathrm{EE}}$	0.082	0.137		
***Matrix***	***0.045***	***0.191***	***0.110***	***Error***	***0.084***	***0.146***		

### Three-way Interactions Related to the Substantial Two-way Interactions

**Fig. 1:**
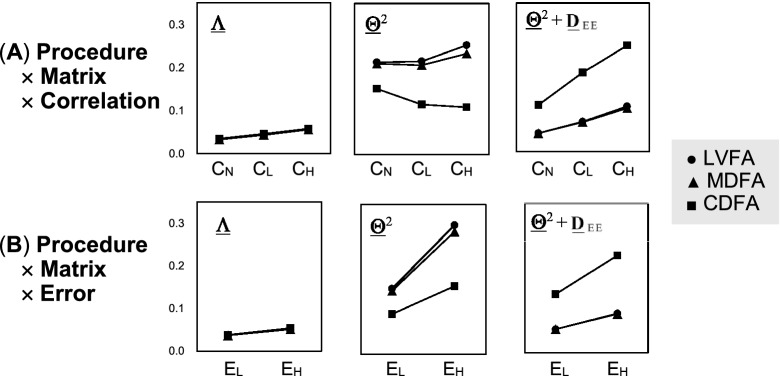
Averages of MAD for three-way interactions (A) and (B), which facilitate the interpretation of the substantial two-way interactions.

Figure 1 shows the averages associated with the P $\times$ M $\times$ C and P $\times$ M $\times$ E interactions, whose *F* values are the largest among those for the three-way interactions (Table 2). Although those values are not substantial, we note Fig. 1 for exploring the mechanisms that underlie the interactions treated in Sect. 5.3.

Panel (A) in Fig. 1 shows that the changes of C$_{\mathrm{N}}$ to C$_{\mathrm{L}}$ and C$_{\mathrm{L}}$ to C$_{\mathrm{H}}$ increase MAD $(\underline{{\varvec{\Lambda }}})$ for all procedures, but decrease the (40) value for CDFA. This result can be explained by the similarity of the matrices ${{\underline{\mathbf{C}}}}_{\mathrm{EE}} =n^{-1}\sum \nolimits _{i=1}^n {{\mathbf{e}}_{i} {{\mathbf{e}'}}_{i} }$ at C$_{\mathrm{N}}$ to (15) and the deviation of ${{\underline{\mathbf{C}}}}_{\mathrm{EE}}$ at C$_{\mathrm{H}}$ from (15). Here, (15) leads to the solution of ${\varvec{\Lambda }} =$ $\underline{{\varvec{\Lambda }}}$ and ${\varvec{\Psi }}^{2\, }=$ ${\varvec{\Theta }}^{2\, }=$ $\underline{{\varvec{\Theta }}}^{2}+{\underline{{\mathbf{D}}}}_{\mathrm{EE}}$, as shown in Corollaries 1–3. However, for ${{\underline{\mathbf{C}}}}_{\mathrm{EE}}$ deviating from (15), the CDFA estimate of ${\varvec{\Theta }} ^{2\, }$ can approximate $\underline{{\varvec{\Theta }}}^{2}$, as discussed with Theorem 6. Thus, the deviation of ${{\underline{\mathbf{C}}}}_{\mathrm{EE}}$ from (15) increases MAD($\underline{\varvec{\Lambda }})$, but decreases (40) only in CDFA. The above explanation can also be used for the increase in the CDFA average of (41) with the deviation from (15). On the other hand, the deviation is not found to decrease the LVFA and MDFA values of (40). This is congruous with (26) and (32). Further, the LVFA and MDFA averages of (40) rather increase with the change from C$_{\mathrm{L}}$ to C$_{\mathrm{H}}$. These increases may be correlated to those in MAD($\underline{{\varvec{\Lambda }}})$ from C$_{\mathrm{L}}$ to C$_{\mathrm{H}}$, as ${\varvec{\Lambda }}$ is jointly estimated with ${\varvec{\Theta }}^{2}$ and ${\varvec{\Psi }}^{2}$ in (40). The LVFA and MDFA averages of (41) being smaller than those of (40) at all C levels is also congruous with (26) and (32).

Panel (B) shows that the increments in MAD with the change from E$_{\mathrm{L}}$ to E$_{\mathrm{H}}$ differ among the procedures and matrices. For explaining the differences, we must consider error magnitudes (EM) and ${\underline{{\mathbf{D}}}}_{\mathrm{EE}}$ effects, where the EM effect stands for the errors with greater EM disturbing parameter estimation more deeply, and the ${\underline{\mathbf{D}}}_{\mathrm{EE}}$ effect refers to the fact that the diagonal elements of ${\underline{{\mathbf{D}}}}_{\mathrm{EE}}$ for E$_{\mathrm{H}}$ have greater values than those for E$_{\mathrm{L}}$. We can consider that the increase in MAD(${\varvec{\Lambda }} )$ for every procedure follows only from the EM effect, while those in the LVFA and MDFA values of (40) are affected by the EM plus ${\underline{{\mathbf{D}}}}_{\mathrm{EE}}$ effects, because of (26) and (32). The increments in (40) being greater than their CDFA counterpart can be explained with Theorem 6, which shows that (32) is less likely to occur in CDFA for ${{\underline{\mathbf{C}}}}_{\mathrm{EE}}$ deviating from (15). On the other hand, (41) values and their increments for MDFA and LVFA are found to be smaller than their (40) counterparts. This result can be attributable to the fact that (41) is affected only by the EM effect in LVFA and MDFA, as the use of their properties (26) and (32) in (41) allows us to find that MAD($\underline{{\varvec{\Theta }}}^{2\, }+$ ${\underline{{\mathbf{D}}}}_{\mathrm{EE}})$ can approximate zero, but this approximation can be deteriorated by the EM effect.

### Additional Results

The results considered so far show the advantage of CDFA over the two other procedures. Which of those two is better may be answered from the following result: The panels for $\underline{{\varvec{\Theta }}}^{2}$ in Fig. 1 show that the MDFA estimate of ${\varvec{\Theta }}^{2\, }$ is slightly closer to $\underline{{\varvec{\Theta }}}^{2}$ than the LVFA estimate of ${\varvec{\Psi }}^{2\, }$ on average. The generality of this relationship was found: The ${\varvec{\Delta }}$ value, which is defined as the LVFA value of (40) minus its MDFA value, was positive for the 5819 pairs of LVFA–MDFA solutions among the 6000 for all **C**$_{\mathrm{XX}}$. Further, we performed ANOVA-RBD for ${\varvec{\Delta }}$ with C, E, and V treated as treatments, and R treated as blocks. Among all resulting *F* values, the one for the main effect of C (3651.7) and the value for the effect of E (3238.4) were the largest, with the other *F* values less than 940. However, the two effects for the largest *F* values are not considered substantial, with the averages at levels C$_{\mathrm{N}}$, C$_{\mathrm{L}}$, C$_{\mathrm{H}}$, E$_{\mathrm{L}}$, and E$_{\mathrm{H}}$ being 0.003, 0.008, 0.019, 0.006, and 0.014, respectively; even the largest of those cannot be regarded as substantial. In conclusion, the MDFA estimate of ${\varvec{\Theta }}^{2}$ is significantly closer to $\underline{{\varvec{\Theta }}}^{2}$ than the LVFA estimate of ${\varvec{\Psi }}^{2}$, but not substantially closer.

## Real Data Illustration

The results in the previous sections can be summarized as follows: In CDFA whose formulation is exactly matched to the CompFA model, its parameters (factor loadings and specific variances) are well recovered for the CompFA data. In MDFA and LVFA, the loadings can be recovered as well as in CDFA, but the specific variances cannot be recovered, so their MDFA estimates are contaminated by the error variances, and the LVFA estimates of the unique variances approximate the sums of the specific and error variances. These results suggest that CDFA should be used for the CompFA data, particularly for the purpose of estimating the specific variances. Therefore, in this section, we use two real data sets to illustrate how the CDFA estimates should be interpreted, and we show the merits of using CDFA through comparisons among CDFA, MDFA, and LVFA solutions, on the supposition that the data sets are underlaid by the CompFA model. Here, LVFA is restricted to ML-LVFA, as its solutions were almost equivalent to LS-LVFA.

One of the data sets is that from Yanai and Ichikawa (2007) for personality test scores of $n =$ 200 students for $p =$ 12 items. The other data set, which we obtained from Izenman’s (2008) website, is known as Holzinger and Swineford’s (1939) 24 psychological tests data and contains intelligence test scores of $n=301$ participants for $p =24$ items. We performed the FA procedures for the correlation matrices for the personality and intelligence test scores, with *m* set at 3 and 5, respectively. In every procedure, ${\varvec{\Lambda }}$ was rotated by the varimax method (Kaiser, 1958) which is typically used in FA for real data. Tables 4 and 5 present the resulting ${\varvec{\Lambda }} =[ {\varvec{\lambda }}_{1},...,{\varvec{\lambda }}_{p}],\Vert {\varvec{\lambda }} _{1}\Vert ^{2},...,\Vert {\varvec{\lambda }}_{p}\Vert ^{2},{\psi }_{j}, {\theta }_{j}$, and *ev*$_{j}$, with the last three terms being the *j*th diagonal elements of ${\varvec{\Psi }}^{2}$, ${\varvec{\Theta }}^{2}$, and **C**$_{\mathrm{EE}}$, respectively, and *ev* an abbreviation of error variance. In the tables, the sub-/superscripts of L, M, and C, which stand for LVFA, MDFA, and CDFA, respectively, have been attached to ${\varvec{\Lambda }}$, ${\varvec{\lambda }}_{j}$, ${\theta }_{j}$, and *ev*$_{j}$, for distinguishing the solutions for different procedures. We use the above notation with sub-/superscripts in this section.

Tables 4 and 5 show that the loadings resulting in all procedures are mutually similar and lead to the identical interpretation of common factors. Although this result does not show the merits of using CDFA, the merits are shown by the other results, as described in the following paragraphs. There, an important role is fulfilled by the fact that

42 $$\begin{aligned} v_{j\, }=\left\| {{\varvec{\lambda }}_{j}^{\mathrm{C}} } \right\| ^{2}+{\varvec{\Theta }}_{j}^{{\mathrm{C}}\;2} +ev_{j}^{\mathrm{C}} =\left\| {{\varvec{\lambda }}_{j}^{\mathrm{M}} } \right\| ^{2}+{\varvec{\Theta }}_{j}^{{\mathrm{M}}\;2} +ev_{j}^{\mathrm{M}} =\left\| {{\varvec{\lambda }}_{j}^{\mathrm{L}} } \right\| ^{2}+{\varvec{\Psi }}_{j}^{2} \end{aligned}$$

holds for $v_{j} = n^{-1}\Vert \mathbf{X }\Vert ^{2}$, which denotes the variance for variable *j* and is also the *j*th diagonal element of **C**$_{\mathrm{XX}}$. Here, the first identity follows from the fact that the CDFA solution meets (9)–(12) and thus (13), the last identity follows from (25), and the second identity is derived as follows: (4), (10), and (11) imply diag(**C**$_{\mathrm{XX}}) =$ diag( ${\varvec{\Lambda }} \varvec{\Lambda }^{\prime } +$ ${\varvec{\Theta }}^{2\, }+$ **C**$_{\mathrm{EE}} + n^{-1} {\varvec{\Theta }}$ **S**$^\prime$ **E** $+ n^{-1}$ **E**$^\prime$ **S**${\varvec{\Theta }} )$ with diag(**E**$^\prime$ **S**${\varvec{\Theta }} ) =$ diag(${\varvec{\Theta }}$ **S**$^\prime$ **E**) $=$ ${\varvec{\Theta }}$diag(**S**$^\prime$ **E**) $=_{\,p}$ **O**$_{p}$ from (28). Each of the terms $\Vert {\varvec{\lambda }}_{j}\Vert ^{2}$ (with superscripts) in (42) can be called a *common variance* (or communality) by abbreviating the variance of the common factor part affecting variable *j*, as found from $\Vert {\varvec{\lambda }}_{j}\Vert ^{2}$ equaling $n^{-1}\Vert \mathbf{F }{\varvec{\lambda }}_{j}\Vert ^{2}$ and the variance of ${\varvec{\lambda }}_{j}^\prime$ **f** under (6) and (10). When **C**$_{\mathrm{XX}}$ is a correlation matrix as in our case, $v_{j} =$ 1; thus, $\Vert {\varvec{\lambda }}_{j}\Vert ^{2}$, ${{\theta }}_{j}^{2}$, and *ev*$_{j}$ (with superscripts) in (42) stand for the proportions of the common, specific, and error variances in the variance of variable *j*, respectively, with ${{\psi }}_{j}^{2}$ the proportion of the unique variance.

**Table 4:** Solutions for personality test data.

Variable	LVFA						MDFA							CDFA					
	${\varvec{\Lambda }}_{\mathrm{L}}=[{\varvec{\lambda }}_{1}^{\mathrm{L}} \cdots {\varvec{\lambda }}_{12}^{\mathrm{L}} {]}'$			$\left\| {{\varvec{\lambda }}_{j}^{\mathrm{L}} } \right\| ^{2}$	${\psi }_{j}^{2}$		${\varvec{\Lambda }}_{\mathrm{M}}=[{\varvec{\lambda }}_{1}^{\mathrm{M}} \cdots {\varvec{\lambda }}_{12}^{\mathrm{M}} {]}'$			$\left\| {{\varvec{\lambda }}_{j}^{\mathrm{M}} } \right\| ^{2}$	${\theta }_{j}^{{\mathrm{M}}2}$	$ev_{j}^{\mathrm{M}}$		${\varvec{\Lambda }}_\mathrm{C\, }=[{\varvec{{\varvec{\Lambda }}}}_{1}^{\mathrm{C}} \cdots {\varvec{\lambda }}_{12}^{\mathrm{C}} {]}'$			$\left\| {{\varvec{\lambda }}_{j}^{\mathrm{C}} } \right\| ^{2}$	${\theta }_{j}^{{\mathrm{C}}2}$	$ev_{j}^{\mathrm{C}}$
*Extraversion*	− 0.32	0.48	0.24	0.39	0.61		− 0.34	0.46	0.25	0.39	0.60	0.011		− 0.35	0.50	0.25	0.44	0.39	0.176
*Activity*	− 0.25	0.65	0.42	0.66	0.33		− 0.26	0.64	0.43	0.66	0.33	0.007		− 0.25	0.67	0.43	0.70	0.18	0.118
*Empathy*	− 0.02	0.03	0.58	0.34	0.66		− 0.03	0.01	0.60	0.36	0.62	0.018		− 0.04	0.00	0.63	0.40	0.45	0.151
*Novelty*	− 0.05	0.62	0.04	0.39	0.62		− 0.06	0.61	0.05	0.38	0.61	0.006		− 0.06	0.65	0.04	0.43	0.44	0.141
*Durability*	− 0.07	0.04	0.68	0.47	0.53		− 0.07	0.04	0.67	0.46	0.54	0.014		− 0.06	0.05	0.68	0.47	0.38	0.151
*Regularity*	0.06	0.17	0.71	0.54	0.46		0.07	0.17	0.71	0.54	0.45	0.015		0.09	0.18	0.76	0.62	0.23	0.152
*Self-revelation*	0.16	0.62	0.03	0.41	0.59		0.14	0.62	0.04	0.41	0.58	0.006		0.16	0.66	0.03	0.46	0.42	0.122
*Aggressiveness*	0.39	0.54	− 0.14	0.46	0.54		0.39	0.57	− 0.15	0.50	0.49	0.010		0.41	0.56	− 0.17	0.51	0.41	0.084
*Uncooperativeness*	0.45	0.16	− 0.17	0.26	0.74		0.46	0.18	− 0.19	0.28	0.71	0.020		0.51	0.20	− 0.23	0.35	0.39	0.259
*Inferiority feeling*	0.62	− 0.30	− 0.17	0.50	0.49		0.63	− 0.29	− 0.17	0.51	0.48	0.007		0.63	− 0.30	− 0.17	0.52	0.37	0.111
*Nervousness*	0.71	0.00	0.22	0.55	0.45		0.70	0.01	0.23	0.54	0.44	0.011		0.76	− 0.02	0.27	0.65	0.18	0.169
*Depression*	0.82	0.03	−0.07	0.68	0.32		0.82	0.04	− 0.06	0.68	0.32	0.007		0.82	0.03	− 0.07	0.68	0.22	0.103

**Table 5:** Solutions for intelligence test data.

Variable	LVFA							MDFA								CDFA							
	${\varvec{\Lambda }}_{\mathrm{L}}=[{\varvec{\lambda }}_{1}^{\mathrm{L}} \cdots {\varvec{\lambda }}_{24}^{\mathrm{L}} {]}'$					$\left\| {{\varvec{\lambda }}_{j}^{\mathrm{L}} } \right\| ^{2}$	${\psi }_{j}^{2}$	${\varvec{\Lambda }}_{{\mathrm{M}}}=[{\varvec{\lambda }}_{1}^{\mathrm{M}} \cdots {\varvec{\lambda }}_{24}^{\mathrm{M}} {]}'$					$\left\| {{\varvec{\lambda }}_{j}^{\mathrm{M}} } \right\| ^{2}$	${\theta }_{j}^{{\mathrm{M}}2}$	$ev_{j}^{\mathrm{M}}$	${\varvec{\Lambda }}_{\mathrm{C}}=[{\varvec{\lambda }}_{1}^{\mathrm{C}} \cdots {\varvec{\lambda }}_{24}^{\mathrm{C}} {]}'$					$\left\| {{\varvec{\lambda }}_{j}^{\mathrm{C}} } \right\| ^{2}$	${\theta }_{j}^{{\mathrm{C}}2}$	$ev_{j}^{\mathrm{C}}$
*Spatial 1*	0.31	0.63	0.10	0.12	0.01	0.52	0.49	0.31	0.63	0.10	0.12	0.03	0.52	0.48	0.006	0.30	0.65	0.10	0.13	0.12	0.55	0.35	0.095
*Spatial 2*	0.14	0.48	$-$ 0.06	0.01	0.12	0.27	0.73	0.14	0.48	$-$ 0.06	0.00	0.14	0.27	0.73	0.002	0.13	0.46	$-$ 0.07	0.01	0.22	0.28	0.65	0.075
*Spatial 3*	0.19	0.43	0.06	$-$ 0.03	0.00	0.23	0.77	0.19	0.44	0.07	$-$ 0.03	0.01	0.24	0.76	0.005	0.19	0.49	0.06	$-$ 0.03	0.05	0.28	0.54	0.177
*Spatial 4*	0.06	0.63	0.09	0.14	0.09	0.44	0.56	0.06	0.64	0.09	0.14	0.10	0.45	0.55	0.002	0.05	0.61	0.08	0.15	0.20	0.44	0.49	0.059
*Verbal 1*	0.82	0.06	0.12	$-$ 0.02	0.04	0.69	0.30	0.83	0.06	0.12	$-$ 0.02	0.04	0.71	0.29	0.008	0.84	0.07	0.11	$-$ 0.02	0.06	0.73	0.16	0.106
*Verbal 2*	0.80	0.10	0.09	0.12	0.06	0.68	0.32	0.80	0.10	0.09	0.12	0.07	0.68	0.32	0.007	0.80	0.10	0.08	0.12	0.11	0.68	0.23	0.087
*Verbal 3*	0.88	0.05	0.06	0.01	$-$ 0.02	0.78	0.22	0.89	0.04	0.06	0.01	0.00	0.80	0.20	0.007	0.91	0.04	0.06	0.00	0.04	0.83	0.07	0.104
*Verbal 4*	0.71	0.16	0.10	0.10	0.07	0.55	0.44	0.71	0.15	0.10	0.10	0.10	0.56	0.44	0.007	0.72	0.14	0.10	0.10	0.15	0.58	0.29	0.139
*Verbal 5*	0.82	0.15	0.06	0.08	0.11	0.72	0.28	0.82	0.15	0.05	0.08	0.12	0.72	0.28	0.007	0.83	0.14	0.05	0.08	0.16	0.74	0.15	0.114
*Speed 1*	0.08	$-$ 0.08	0.79	0.08	0.14	0.66	0.34	0.08	$-$ 0.09	0.78	0.08	0.14	0.65	0.34	0.010	0.07	$-$ 0.12	0.80	0.07	0.14	0.68	0.23	0.098
*Speed 2*	0.29	0.18	0.57	0.24	$-$ 0.09	0.51	0.50	0.30	0.18	0.57	0.23	$-$ 0.04	0.50	0.49	0.009	0.30	0.17	0.60	0.23	0.02	0.53	0.31	0.155
*Speed 3*	0.09	0.23	0.62	0.00	0.04	0.45	0.56	0.09	0.22	0.62	0.00	0.06	0.44	0.55	0.005	0.08	0.22	0.65	0.00	0.11	0.49	0.37	0.137
*Speed 4*	0.17	0.45	0.51	0.06	−0.11	0.51	0.50	0.17	0.44	0.51	0.05	$-$ 0.06	0.49	0.51	0.007	0.16	0.46	0.54	0.04	0.03	0.53	0.32	0.142
*Memory 1*	0.15	0.05	0.04	0.64	0.08	0.44	0.56	0.15	0.04	0.04	0.63	0.11	0.43	0.56	0.007	0.15	0.00	0.02	0.69	0.14	0.52	0.31	0.179
*Memory 2*	$-$0.03	0.16	0.05	0.56	0.08	0.35	0.66	$-$ 0.03	0.17	0.04	0.57	0.08	0.36	0.63	0.010	$-$ 0.05	0.17	0.02	0.63	0.09	0.44	0.39	0.178
*Memory 3*	0.17	0.37	0.11	0.45	0.18	0.41	0.58	0.17	0.36	0.11	0.44	0.22	0.41	0.58	0.006	0.16	0.32	0.10	0.44	0.30	0.42	0.48	0.105
*Memory 4*	0.06	0.03	0.34	0.54	$-$ 0.03	0.41	0.60	0.06	0.03	0.34	0.54	$-$ 0.02	0.41	0.58	0.007	0.06	0.02	0.35	0.57	$-$ 0.01	0.45	0.39	0.153
*Memory 5*	0.12	0.17	0.22	0.42	0.01	0.27	0.73	0.12	0.17	0.23	0.44	0.02	0.29	0.70	0.012	0.12	0.17	0.24	0.49	0.02	0.34	0.43	0.226
*Memory 6*	0.24	0.16	0.11	0.31	0.22	0.24	0.76	0.24	0.13	0.12	0.30	0.26	0.25	0.74	0.013	0.23	0.06	0.12	0.31	0.35	0.29	0.51	0.200
*Math.1*	0.34	0.39	$-$ 0.01	0.21	0.31	0.41	0.60	0.33	0.38	$-$ 0.01	0.21	0.32	0.40	0.60	0.004	0.31	0.33	$-$ 0.03	0.21	0.40	0.41	0.49	0.101
*Math.2*	0.29	0.35	0.35	0.10	0.39	0.49	0.52	0.28	0.34	0.34	0.10	0.42	0.50	0.50	0.007	0.24	0.26	0.33	0.08	0.55	0.54	0.32	0.133
*Math.3*	0.49	0.36	0.06	0.11	0.24	0.44	0.55	0.49	0.35	0.06	0.11	0.27	0.45	0.55	0.009	0.48	0.29	0.05	0.10	0.38	0.47	0.38	0.155
*Math.4*	0.42	0.47	0.16	0.13	0.36	0.57	0.43	0.41	0.46	0.15	0.12	0.38	0.56	0.43	0.008	0.39	0.40	0.13	0.11	0.51	0.60	0.23	0.160
*Math.5*	0.42	0.12	0.36	0.19	0.42	0.53	0.47	0.41	0.09	0.35	0.18	0.47	0.55	0.45	0.005	0.40	0.01	0.34	0.17	0.51	0.56	0.35	0.085

Let us note the CDFA solution in Table 4 with keeping (42) in mind. For example, the results $\left\| {{\varvec{\lambda }}_{1}^{\mathrm{C}} } \right\| ^{2}=$ 0.44, ${\varvec{\Theta }}_{1}^{{\mathrm{C}}2} =$ 0.39, and $ev_{1}^{\mathrm{C}} =$ 0.176 for the variable *extraversion* show that 44 percent of the variations in *extraversion* are explained by the common factors, 39 percent are accounted for by the factor specific to *extraversion*, and 17.6 percent of the variations remain unexplained by common and specific factors. The comparison of ${\varvec{\Theta }}_{j}^{{\mathrm{C}}2}$ across *j* ($=$ 1, …, *p*) shows that ${\varvec{\Theta }}_{3}^{{\mathrm{C}}2} =$ 0.45 for *empathy *is the largest and *empathy *is affected most by the corresponding specific factor among all variables. On the other hand, ${\varvec{\Theta }}_{2}^{{\mathrm{C2}}} =$ 0.18 for *activity* is the smallest among all ${\varvec{\Theta }}_{j}^{{\mathrm{C}}2}$, but $\left\| {{\varvec{\lambda }}_{2}^{\mathrm{C}} } \right\| ^{2}=$ 0.70 is the largest among all $\left\| {{\varvec{\lambda }}_{j}^{\mathrm{C}} } \right\| ^{2}$, implying that *activity* is affected least by the corresponding specific factor, but is explained best by the common factors, among all variables.

MDFA and LVFA solutions can be interpreted in a parallel manner, except that the term "specific" is replaced by "unique" and an error variance is not obtained in LVFA. However, we can find in Tables 4 and 5 that ${\varvec{\Theta }} _{j}^{\mathrm{M2}}$ and ${\varvec{\Psi }}_{j}^{2}$ are much greater than ${\varvec{\Theta }}_{j}^{{\mathrm{C}}2}$ and rather close to ${\varvec{\Theta }}_{j}^{{\mathrm{C}}2} +ev_{j}^{\mathrm{C}}$ for almost all variables. This finding is congruous with (26) and (32). For example, ${\varvec{\Theta }}_{1}^{{\mathrm{C}}2} =$ 0.60 and ${\varvec{\Psi }}_{1}^{2} =$ 0.61 > ${\varvec{\Theta }}_{1}^{{\mathrm{C}}2} =$ 0.39 for the variable *extraversion* in Table 4. We can consider that ${\varvec{\Theta }}_{1}^{{\mathrm{C}}2} =$ 0.60 and ${\varvec{\Psi }}_{1}^{2} =$ 0.61 are contaminated by the true error variance for *extraversion* and greater than its true specific variance.

We can also find that the error variance $ev_{j}^{\mathrm{M}}$ resulting in MDFA is far smaller than its CDFA counterpart $ev_{j}^{\mathrm{C}}$ for every variable. This result follows from the fact that MDFA is less restrictive than CDFA; (27) value $n^{-1}\Vert \mathbf{E }\Vert ^{2} =$ tr**C**$_{\mathrm{EE}} =\sum \nolimits _{j=1}^p {ev_{j} }$ in MDFA cannot be greater than that in CDFA, which suggests that $ev_{j}^{\mathrm{M}}$ < $ev_{j}^{\mathrm{C}}$ tends to occur. This property does not imply goodness of the MDFA solution but is rather congruous with its undesirable property shown by (32); comparing this with $ev_{j}^{\mathrm{M}} = v_{j} -\left\| {{\varvec{\lambda }}_{j}^{\mathrm{M}} } \right\| ^{2}-{\varvec{\Theta }}_{j}^{{\mathrm{M}}2}$ following from (42) allows us to find that ${\varvec{\Theta }}_{j}^{{\mathrm{M}}2}$ being larger than its true value decreases $ev_{j}^{\mathrm{M}}$.

## Conclusion

In this paper, latent variable factor analysis (LVFA), matrix decomposition factor analysis (MDFA), and its variant from Stegeman (2016) were revisited from the comprehensive FA (CompFA) model. The variant of MDFA was reformulated to be called CDFA and exactly underlaid by the CompFA model. On the other hand, MDFA was reformulated as the procedure with (12) in the CompFA model assumptions relaxed as (28). We also showed how the model for LVFA can be related to the CompFA model.

A goal of the revisit was to show how LVFA, MDFA, and CDFA behave for the CompFA data based on the CompFA model. Except for the unusual case where the data satisfy strong condition (15), the following results were theoretically and numerically found: The CDFA estimates of the specific variances can approximate their true values, but the MDFA estimates are contaminated by the error variances, and the LVFA estimates of the unique variances approximate the sum of the true specific and error variances. It was also shown numerically that the factor loadings can be recovered well in all three procedures. On the supposition that the data to be analyzed by FA are underlaid by the CompFA model, the above results have the practical implications described in the following paragraph.

When only factor loadings are of interest, LVFA, MDFA, and CDFA are equally useful. However, if the specific variances are also interesting, CDFA is to be used. The LVFA estimate of unique variances and the MDFA estimates of specific variances must be considered as larger than the true specific variances.

However, a problem remains for showing the above implications to FA users, who are interested in only the factor loadings, not the specific variances. This problem would be dealt with, if psychometricians could enlighten the users about the importance of the specific variances. For this enlightenment, CompFA model (4) can be used as follows: By removing the specific factor part by setting ${\varvec{\Theta }} =$ $_{p}$ **O**$_{p\, }$ in (4), this model and corresponding least squares function (27) are rewritten as **X** $=$ **F**${\varvec{\Lambda }}^{\prime } +$ **E** and $\Vert \mathbf{X }-\mathbf{F} {\varvec{\Lambda }}^{\prime } \Vert ^{2}$, respectively. Minimizing this function gives the formulation of principal component analysis (PCA) as approximating **X** by reduced rank matrix **F**${\varvec{\Lambda }}^{\prime }$ (Eckart & Young, 1936). This fact demonstrates that FA can be distinguished from PCA simply by the fact that the former has specific factor part **S**${\varvec{\Theta }}$. That is, the significance of using FA (rather than PCA) is in obtaining **S**${\varvec{\Theta }}$, which convinces the users of how it is necessary to interpret the specific variances in ${\varvec{\Theta }}^{2}$ together with loading matrix ${\varvec{\Lambda }}$ in the FA solution.

Finally, we should remember that MDFA can be regarded as a procedure for a relaxed variant of the CompFA model with (12) replaced by (28). To study such a relaxed CompFA model is beyond the scope of the present study; thus, it remains for future approaches.

## Appendix 1.Expectation of the Error Covariance Matrix

If the *n* rows of **E** in (4) are filled with the realizations of the transpose of **e** in (3), the expectation of **C**$_{\mathrm{XX}} =$ $n^{-1}$ **E**$^\prime$ **E** is rewritten as

$$\begin{aligned} E[\mathbf{C }_{\mathrm{XX}}] =E\left[ {\frac{1}{n}{{\mathbf{E}'{\mathbf{E}}}}} \right] =E\left[ {\frac{1}{n}\sum \limits _{i=1}^n {{{{\mathbf{e}}{\mathbf{e}}'}}} } \right] = E[\mathbf{ee} ^\prime ] = E[(\mathbf{e }- E[\mathbf{e }])(\mathbf{e }- E[\mathbf{e }])^\prime ] = C[\mathbf{e }, \mathbf{e }]. \end{aligned}$$

Here, we have used (5).

## Appendix 2. Proof for Theorem 3.

In the proof, we use the fact that ${{\underline{\mathbf{F}}}}$, ${{\underline{\mathbf{S}}}}$, and ${\underline{\mathbf{E}}}$ in (17) can be substituted into **F**, **S**, and **E** in (9)–(12), respectively:

A1 $$\begin{aligned} {{\mathbf{1}}}_{n}' {\underline{{\mathbf{F}}}}=\mathbf{0}_{m}^\prime , {{\mathbf{1}}}_{n}' {\underline{{\mathbf{S}}}}= \mathbf{0 }_{p}^\prime , {{\mathbf{1}}}_{n}' {\underline{{\mathbf{E}}}}= \mathbf{0 }_{p}^\prime , \frac{1}{n}{{{\underline{\mathbf{F}}}'\underline{\mathbf{F}}}}= \mathbf{I }_{m} , \frac{1}{n}{{{\underline{\mathbf{S}}}'\underline{\mathbf{S}}}}= \mathbf{I }_{p\, },\nonumber \\ {{\underline{{\mathbf{F}}}'\underline{\mathbf{S}}}}=_{\,m}\!\!\mathbf{O }_{p} , {{\underline{{\mathbf{F}}}'\underline{\mathbf{E}}}} =_{\,m}\!\!\mathbf{O }_{p\, }, {{\underline{{\mathbf{S}}}'\underline{\mathbf{E}}}}={}_{\,p} {\mathbf{O}}_{p} . \end{aligned}$$

We also use that fact that (29) and rank$({\underline{{\mathbf{E}}}})= p$ imply the nonsingularity of $\underline{\varvec{\Gamma }}^{\prime }$ and ${\underline{{\mathbf{G}}}}={{\underline{\mathbf{E}}\,{\underline{{\varvec{\Gamma }}}}'}}^{-1}$. This equation and (A1) imply

A2 $$\begin{aligned} {{\mathbf{1}}}_{n}' {\underline{{\mathbf{G}}}}=\mathbf{0}_{p}^\prime ,\, {{\underline{{\mathbf{F}}}'\underline{\mathbf{G}}}} =_{\,m}\!\!\mathbf{O }_{p},\,\, \hbox {and}\,\, {{\underline{{\mathbf{S}}}'\underline{\mathbf{G}}}}=_{\,p}\!\!\mathbf{O }_{p} . \end{aligned}$$

At first, ${{\bar{\varvec{\Theta }}}}$ and ${{\bar{\mathbf{S}}}}$ in (30) can be substituted into ${\varvec{\Theta }}$ and **S** in (27), respectively, since ${{\bar{\varvec{\Theta }}}}=$ $\underline{{\varvec{\Theta }}}^{2}+ {\varvec{\Delta }} ^{2})^{1/2\, }$ is a *p* $\times$ *p* diagonal matrix as is ${\varvec{\Theta }}$, and ${{\bar{\mathbf{S}}}} =$ (${{\underline{\mathbf{S}}\,\underline{{\varvec{{\Theta }}}}}}+{{\underline{\mathbf{G}}{\varvec{\Delta }} }}){{\bar{\varvec{\Theta }}}}^{-1}$ is *n* $\times$ *p* as is **S**. The substitution of ${{\bar{\mathbf{S}}}}=$ (${{\underline{\mathbf{S}}\underline{{\varvec{{\Theta }}}}}}+{{\underline{\mathbf{G}}{\varvec{\Delta }} }}){{\bar{\varvec{\Theta }}}}^{-1}$ into **S** in **1**$_{n}^\prime$ **S** and $n^{-1}$ **S**$^\prime$ **S** leads to **1**$_{n}^\prime$ **S**$=$ **0**$_{p}^\prime$ in (9) and $n^{-1}$ **S**$^\prime$ **S** $=$ **I**$_{p}$ in (10) as follows: **1**$_{n}^\prime$ **S**$=$ (${{\mathbf{1}'}}_{n} {{\underline{\mathbf{S}}\,\underline{{\varvec{{\Theta }}}}}}+{{\mathbf{1}'}}_{n} {{\underline{\mathbf{G}}{\varvec{\Delta }} }}){{\bar{\varvec{\Theta }}}}^{-1}=$ **0**$_{p}^\prime$ and

$$\begin{aligned} \frac{1}{n}{{\mathbf{S}'{\mathbf{S}}}}= \frac{1}{n} {{\bar{\varvec{\Theta }}}}^{-1}({{\underline{\mathbf{S}}\,\underline{{\varvec{{\Theta }}}}}}+{{\underline{\mathbf{G}}{\varvec{\Delta }} }})^\prime ({{\underline{\mathbf{S}}\,\underline{{\varvec{{\Theta }}}}}}+{{\underline{\mathbf{G}}\,{\varvec{\Delta }} }}){{\bar{\varvec{\Theta }}}}^{-1}=^{\, }(\underline{{\varvec{\Theta }}}^{2\, }+ {\varvec{\Delta }} ^{2})^{-1/2}(\underline{{\varvec{\Theta }}}^{2}+ {\varvec{\Delta }} ^{2})(\underline{{\varvec{\Theta }}}^{2}+ {\varvec{\Delta }} ^{2})^{-1/2\, }= \mathbf{I }_{p}. \end{aligned}$$

Here, we have used (A1), (A2), (29), (30), and rank$({{\varvec{\underline{\Theta }}}})= p$.

Through the following five steps in this paragraph, we show that ${{\bar{\mathbf{S}}}}$ in (30) can be substituted into **S** in (28) for ${\varvec{\Delta }} =$ diag$(\underline{\varvec{\Gamma }})$ and **F** satisfying ${{\mathbf{F}'\bar{{\mathbf{S}}}}}=_{\, m}$ **O**$_{p}$. First, **E** in (28) refers to that in model (4), which shows that **E** in (28) is rewritten as **E** $=$ **X**−**F**${\varvec{\Lambda }}^{\prime } - \mathbf{S }{\varvec{\Theta }}$. Since **X** is specified as (17), this use in **E** $=$ **X** − **F**${\varvec{\Lambda }}^{\prime } -$ **S**${\varvec{\Theta }}$ allows it to be further rewritten as

A3 $$\begin{aligned} \mathbf{E }= ({{\underline{\mathbf{F}}\,{\underline{{\varvec{{\Lambda }}}}}'}}+{{\underline{\mathbf{S}}\,\underline{{\varvec{{\Theta }}}}}}+{{\underline{\mathbf{E}}}}) -{\mathbf{F}}{\varvec{\Lambda }}^{\prime } -\mathbf{S }{\varvec{\Theta }} . \end{aligned}$$

Second, the substitution of ${{\bar{\mathbf{S}}\bar{{{\varvec{\Theta }}}}}}={{\underline{\mathbf{S}}\,\underline{{\varvec{\Theta }}}}}+{{\underline{\mathbf{G}}{\varvec{\Delta }} }}$ following from (30) into **S**${\varvec{\Theta }}$ in (A3) gives

A4 $$\begin{aligned} \mathbf{E }={{\underline{\mathbf{F}}\,{\underline{{\varvec{{\Lambda }}}}}'}}+{{\underline{\mathbf{S}}\,\underline{{\varvec{{\Theta }}}}}}+{{\mathbf{E}}} -{\mathbf{F}}{\varvec{\Lambda }}^{\prime } - ({{\underline{\mathbf{S}}\underline{{\varvec{{\Theta }}}}}}+{\underline{\mathbf{G}}{\varvec{\Delta }}}) ={{\underline{\mathbf{F}}{\underline{{\varvec{{\Lambda }}}}}'}} -{\mathbf{F}}{\varvec{\Lambda }}^{\prime } +{\underline{{\mathbf{E}}}} -{{\underline{\mathbf{G}}{\varvec{\Delta }} }} . \end{aligned}$$

Third, the transpose of ${{\bar{\mathbf{S}}}}$ in (30) post-multiplied by (A4) can be rewritten as

A5 $$\begin{aligned} {{{\bar{\mathbf{S}}}'{\mathbf{E}}}}= {{{\bar{\mathbf{S}}}}'}({{\underline{\mathbf{F}}\,{\underline{{\varvec{\Lambda }}}}'}}+{{\underline{\mathbf{E}}}}-{{\underline{\mathbf{G}}{\varvec{\Delta }} }}) = {{\bar{\varvec{\Theta }}}}^{-1}({{\underline{\mathbf{S}}\,\underline{{{\varvec{\Theta }}}}}}+{{\underline{\mathbf{G}}{\varvec{\Delta }} }})^\prime ({{\underline{\mathbf{F}}{\underline{{{\varvec{\Lambda }}}}}'}}+{{\underline{\mathbf{E}}}} -{{\underline{\mathbf{G}}{\varvec{\Delta }} }}) , \end{aligned}$$

where the first identity follows from ${{\mathbf{F}'\bar{\mathbf{S}}}}=$ $_{m}$ **O**$_{p}$. Fourth, we can use (A1), (A2), and (29) to further rewrite (A5) as

A6 $$\begin{aligned} {{{\bar{\mathbf{S}}}'{\mathbf{E}}}}= & {} {{\bar{\varvec{\Theta }}}}^{-1}{{{\varvec{\Delta }}{\underline{\mathbf{G}}}'}}({{\underline{\mathbf{F}}\,{\underline{{{\varvec{\Lambda }}}}}'}}+{{\underline{\mathbf{E}}}}-{{\underline{\mathbf{G}}{\varvec{\Delta }}}})\nonumber \\= & {} {{\varvec{\Theta }}}^{-1}({\varvec{\Delta }} {{{\underline{\mathbf{G}}}'\underline{\mathbf{E}}}}- {\varvec{\Delta }} {{\underline{{\mathbf{G}}}'\underline{\mathbf{G}}}}{\varvec{\Delta }} )\nonumber \\= & {} {{\bar{\varvec{\Theta }}}}^{-1}({\varvec{\Delta }} {{\underline{{\mathbf{G}}}'\,\underline{\mathbf{G}}}}\varvec{\Gamma }^{\prime } -{\varvec{\Delta }} \underline{{\mathbf{G}}}'\underline{\mathbf{G}}{\varvec{\Delta }})\nonumber \\= & {} n{{\bar{\varvec{\Theta }}}}^{-1}({\varvec{\Delta }} \underline{\varvec{\Gamma }}^{\prime } - {\varvec{\Delta }}^{2}) . \end{aligned}$$

Finally, the substitution of (A6) into **S**$^\prime$ **E** in (28) leads to $\hbox {diag}({{{\bar{\mathbf{S}}}'{\mathbf{E}}}})=n \hbox {diag}({{\bar{\varvec{\Theta }}}}^{-1} ({{{\varvec{\Delta }}{\underline{{\varvec{\varvec{\Gamma }}}}'}}} -{\varvec{{\varvec{\Delta }}}}^{2}))\,n {{\bar{\varvec{\Theta }}}}^{-1} \hbox {diag}({{{\varvec{\Delta }}{\underline{{\varvec{\varvec{\Gamma }}}}'}}}- {\varvec{{\varvec{\Delta }}}}^{2})=n{{\bar{\varvec{\Theta }}}}^{-1}\{{\varvec{{\varvec{\Delta }}}} \hbox {diag}( \underline{{\varvec{\varvec{\Gamma }}}}) -{\varvec{{\varvec{\Delta }}}}^{2}\}=_{\,p}$ **O**$_{p}$ for ${\varvec{\Delta }}$ $=$ diag$(\underline{\varvec{\Gamma }})$.

The remaining task is to show that substituting $${\varvec{\Theta }} $$$$={{\bar{\varvec{\Theta }}}}$$, **S**$$={{\bar{\mathbf{S}}}}$$ and (17) in (27) allows this function to be rewritten as (31), for $${\varvec{\Delta }} =$$ diag$$(\underline{\varvec{\Gamma }})$$ and **F** satisfying **F**$$^\prime {\underline{{\mathbf{E}}}}=_{\,m}$$ **O**$$_{p}$$. Function (27) after the substitution is given by the use of (A4) in (27): *f*(**F**, $${\varvec{\Lambda }} $$, $${{\bar{\mathbf{S}}}}$$, $${{\bar{\varvec{\Theta }}}}) = n^{-1}\Vert {{\underline{\mathbf{F}}{\underline{{{\varvec{\Lambda }} }}}'}} -\mathbf{F }{\varvec{\Lambda }}^{\prime } +{{\underline{\mathbf{E}}}} -\left. {{{\underline{\mathbf{G}}{\varvec{\Delta }} }}} \right\| ^{2}$$. Further, this can be decomposed as

A7 $$\begin{aligned} f({\mathbf{F}}, {\varvec{\Lambda }} , {{\bar{\mathbf{S}}}}, {{\bar{\varvec{\Theta }}}}) =\frac{1}{n}\Vert {{\underline{\mathbf{F}}\,{\underline{{{\varvec{\Lambda }}}}}'}} -{\mathbf{F}}{\varvec{\Lambda }}^{\prime } \Vert ^{2\, }+\frac{1}{n}\Vert {\underline{{\mathbf{E}}}} -{{\underline{\mathbf{G}}{\varvec{\Delta }} }}\Vert ^{2\, \, }, \end{aligned}$$

since (A1), (A2), ${{\mathbf{F}'\underline{\mathbf{E}}}}=_{\,m}$ **O**$_{p}$, and the equation ${{\mathbf{F}'\underline{\mathbf{G}}}}=_{\,m}\!\!\mathbf{O }_{p}$ following from ${{\mathbf{F}'\underline{\mathbf{E}}}}=_{\,m}\!\!\mathbf{O }_{p}$ and ${{\underline{\mathbf{G}}}}={{\underline{\mathbf{E}}\,{\underline{{\varvec{\Gamma }}}}'}}^{-1}$. The last term in (A7) can be rewritten as $n^{-1}\Vert {{\underline{\mathbf{E}}}} -{{\underline{\mathbf{G}}{\varvec{\Delta }} }}\Vert ^{2\, }=\hbox {tr}{{\underline{\mathbf{D}}}}_{\mathrm{EE}} +$ tr${\varvec{\Delta }}^{2}$ $- 2n^{-1}\hbox {tr}{{\mathbf{\underline{\mathbf{E}}}'\underline{\mathbf{G}}{\varvec{\Delta }} }}=\hbox {tr}{{\underline{\mathbf{D}}}}_{\mathrm{EE}} +$ tr${\varvec{\Delta }}^{2} -$ 2tr$\underline{\varvec{\Gamma }}{\varvec{\Delta }}$ $=\hbox {tr}{{\underline{\mathbf{D}}}}_{\mathrm{EE}} +$ tr${\varvec{\Delta }} ^{2} -$ 2tr diag($\underline{\varvec{\Gamma }}) {\varvec{\Delta }} =\hbox {tr}{{\underline{\mathbf{D}}}}_{\mathrm{EE}} -$ tr${\varvec{\Delta }}^{2}$, where we have used $n^{-1}\hbox {tr} {{{\underline{\mathbf{E}}}'\underline{\mathbf{E}}}}=\hbox {tr}{{\underline{\mathbf{D}}}}_{\mathrm{EE}}$, ${\varvec{\Delta }}$ $=$ diag$(\underline{\varvec{\Gamma }})$, (29), and this implying ${{\mathbf{\underline{\mathbf{E}}}'\underline{\mathbf{G}}}} =$ *n* $\underline{\varvec{\Gamma }}$ . This completes the proof for Theorem 3.

## Appendix 3. Demonstration for Corollaries

We performed a simulation study. Its procedures are the same as those in Sects. 5.1 and 6.2, except that simulated data satisfy (15): **C**$_{\mathrm{XX}}$ are restricted to (38) with its ${{\underline{\mathbf{C}}}}_{\mathrm{EE}}$ replaced by the diagonal matrix ${\varvec{\Phi }}$ for the C$_{\mathrm{N}}$ level. For all of the resulting 1000 **C**$_{\mathrm{XX}}$ ($=$ 500 replications $\times$ 2 levels of E$_{\mathrm{L}}$ and E$_{\mathrm{H}})$, every procedure provided the solutions with the loss function value $=$ 0, MAD($\underline{{\varvec{\Lambda }}}$) < 0.0006, and the (41) value < 0.0015.

## Appendix 4. Algorithms

Here, we describe the FA algorithms used in this paper. Harman and Jones’ (1966) MINRES and Adachi’s (2013) version of Rubin and Thayer’s (1982) EM algorithm are used in LS- and ML-LVFA, respectively. Adachi’s (2012, 2020) algorithm is used for MDFA. Bertisimas, Cohenhaver, and Mazumder’s (2017) algorithm is used for the first MRFA step in CDFA, and Stegeman’s (2016) algorithm is used for the remaining steps in CDFA. The above LS-LVFA algorithm can give an improper solution including a negative unique variance; such a solution cannot be given by the algorithms for the other procedures (Adachi, 2013, 2019).
